# Drug design from the cryptic inhibitor envelope

**DOI:** 10.1038/ncomms10638

**Published:** 2016-02-25

**Authors:** Chul-Jin Lee, Xiaofei Liang, Qinglin Wu, Javaria Najeeb, Jinshi Zhao, Ramesh Gopalaswamy, Marie Titecat, Florent Sebbane, Nadine Lemaitre, Eric J. Toone, Pei Zhou

**Affiliations:** 1 Department of Biochemistry, Duke University Medical Center, Durham, North Carolina 27710, USA; 2 Department of Chemistry, Duke University, Durham, North Carolina 27708, USA; 3 Inserm, Univ. Lille, CHU Lille, Institut Pasteur de Lille, CNRS, U1019-UMR 8204-CIIL-Center for Infection and Immunity of Lille, F-59000 Lille, France

## Abstract

Conformational dynamics plays an important role in enzyme catalysis, allosteric regulation of protein functions and assembly of macromolecular complexes. Despite these well-established roles, such information has yet to be exploited for drug design. Here we show by nuclear magnetic resonance spectroscopy that inhibitors of LpxC—an essential enzyme of the lipid A biosynthetic pathway in Gram-negative bacteria and a validated novel antibiotic target—access alternative, minor population states in solution in addition to the ligand conformation observed in crystal structures. These conformations collectively delineate an inhibitor envelope that is invisible to crystallography, but is dynamically accessible by small molecules in solution. Drug design exploiting such a hidden inhibitor envelope has led to the development of potent antibiotics with inhibition constants in the single-digit picomolar range. The principle of the cryptic inhibitor envelope approach may be broadly applicable to other lead optimization campaigns to yield improved therapeutics.

## Abstract (web summary)

The conformational dynamics of a compound has a large effect on ligand/receptor binding. Here, the authors employ NMR spectroscopy to study ligand binding to the enzyme LpxC, discovering an inhibitor envelope that was not identifiable by crystallography and subsequently developing a highly potent inhibitor.

## Graphical abstract

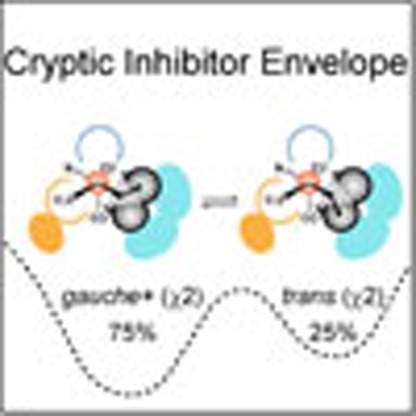

The availability of high-resolution crystal structures of protein-inhibitor complexes has revolutionized the drug development process, enabling structure-aided design of improved therapeutics based on visual inspection of receptor-ligand interactions. However, it is increasingly recognized that high-resolution structures of protein-inhibitor complexes do not necessarily enable a successful lead optimization campaign, as the static structural models often fail to capture the conformational flexibility of receptors or their bound inhibitors^1^,^2^. In contrast to the largely static view of protein structures provided by crystallography, the discovery of ring flipping events of buried aromatic residues of the basic pancreatic trypsin inhibitor by NMR (ref. 3) has heralded the widespread observation of molecular motions within macromolecules in solution. Conformational dynamics involving side-chain rearrangement, domain reorganization and binding-induced structural remodelling has been shown to play important roles in enzyme catalysis^4^,^5^,^6^,^7^, allosteric regulation^8^ and nucleic acid function^9^. Molecular recognition of small molecules likewise alters protein dynamics^10^. Despite the extensive demonstration of conformational dynamics of macromolecules in solution, the application of such information to drug development has remained an unmet challenge.

In this study, we used solution NMR to investigate the conformational states of small-molecule inhibitors bound to LpxC, an essential metalloamidase that catalyses the deacetylation of UDP-(3-*O*-acyl)-*N*-acetylglucosamine during the biosynthesis of lipid A in Gram-negative bacteria^11^,^12^. We show that these enzyme-bound inhibitors dynamically access alternative, minor conformations in solution in addition to the ligand state observed in the crystal structure. Furthermore, we demonstrate that these ligand conformational states collectively define a cryptic inhibitor envelope that can be exploited for optimization of lead compounds.

## Results

### A cryptic inhibitor envelope invisible in crystal structures

**Figure 1:**
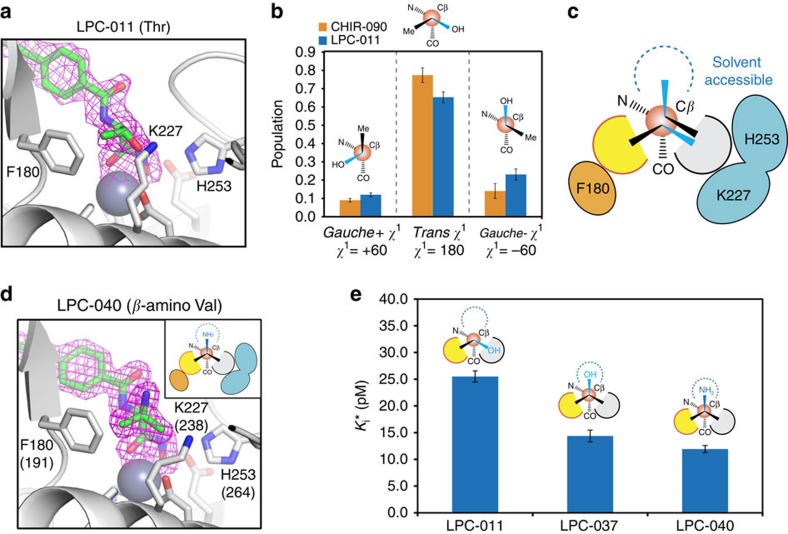
Dynamic access of minor conformational states of LpxC inhibitors containing the threonyl head group. (**a**) Crystal structure of the AaLpxC/LPC-011 complex, showing a single *trans χ*^1^ rotamer of the threonyl side chain of the inhibitor. AaLpxC is shown in the cartoon model and catalytically important residues in the stick model. LPC-011 is shown in the stick model, with the purple mesh representing the inhibitor omit map (2mFo-DFc) contoured at 1.0σ. (**b**) NMR measurements of scalar couplings (^3^*J*_NCγ2_ and ^3^*J*_C'Cγ2_) of the threonyl-head-group-containing LpxC inhibitors CHIR-090 (orange) and LPC-011 (blue) reveal a dynamic distribution of all three rotameric *χ*^1^ states. (**c**) Combining the two most-populated ligand states creates a dynamically accessible inhibitor envelope around the Cβ atom of the threonyl head group. The binding pockets near F180 and H253/K227 are coloured in yellow and grey, respectively, and a third binding pocket accessible to solvent is denoted by an open dashed circle in blue. (**d**) The Cβ-triply substituted compound LPC-040 occupies all three pockets within the inhibitor envelope. PaLpxC is shown in the cartoon model, with catalytically important residues shown in the stick model. Residue numbering reflects the corresponding residues in AaLpxC, with PaLpxC residue numbers shown in parentheses. LPC-040 is shown in the stick model, with the purple mesh representing the inhibitor omit map (2mFo-DFc) contoured at 1.0σ. (**e**) Inhibition constants (*K*_i_*) of LpxC inhibitors. Chemical substitutions at the Cβ-position of the inhibitors and their observed (LPC-011 and LPC-040) and predicted (LPC-037) binding modes within the inhibitor envelope are labelled.

We chose *Aquifex aeolicus* LpxC (AaLpxC) in the lipid A biosynthetic pathway (Supplementary Fig. 1) for structural and dynamics investigation due to its exceptional thermostability, which has enabled both NMR measurements and crystallographic studies (for example, refs 13, 14, 15, 16). *Pseudomonas aeruginosa* LpxC (PaLpxC) was exploited when co-crystal structures of the desired AaLpxC-inhibitor complexes could not be obtained. As a starting point, we investigated the conformations of CHIR-090 and LPC-011 bound to AaLpxC, two inhibitors that share the same threonyl-hydroxamate head group, but differ in their tail groups (Supplementary Fig. 1, Supplementary Table 1). CHIR-090 features a substituted biphenyl acetylene tail group that competes with the acyl chain of the LpxC substrate to occupy the hydrophobic substrate passage of the enzyme^14^. Replacing the tail group of CHIR-090 with a substituted biphenyl diacetylene group generated LPC-011 with improved antibiotic activity due to minimization of vdW clashes with the substrate-binding passage^16^,^17^. To provide a direct comparison with solution NMR investigations, we determined the crystal structure of AaLpxC in complex with LPC-011 (Fig. 1a, Supplementary Table 2). This structure reveals a single conformation of the threonyl-hydroxamate head group in the active site, with the threonyl Cγ2 methyl group packing against an invariant phenylalanine residue (F180 in AaLpxC) and the Oγ1 hydroxyl group forming a hydrogen bond with the catalytically important lysine residue (K227 in AaLpxC). The threonyl side chain of the inhibitor features a *trans* configuration with a *χ*^1^ angle of 180°, a rotameric state that is less energetically favourable (7% population of all threonine side chains in proteins) compared with the alternative rotameric states of *gauche*- (*χ*^1^=−60°) and *gauche+* (*χ*^1^=60°) collectively accounting for 92% of the observed threonine side-chain conformations^18^. Since the observed ligand conformation in the crystal structure represents an unfavourable *χ*^1^ rotameric state of the threonyl head group, we investigated whether this group could access alternative ligand conformations in solution.

Database analysis of high-resolution protein structures has indicated that amino acid side-chains adopt specific rotameric conformations^18^, and side-chain motions can be approximated as conformational hopping between rotameric states^19^. Such motions occur over a wide range of timescales, from ns movement of surface exposed residues to μs-ms timescale ring flipping in protein cores. To determine rotameric populations of the ligand threonyl side chain over a wide timescale, we synthesized isotopically labelled CHIR-090 and LPC-011 and measured the scalar couplings ^3^*J*_NCγ2_ and ^3^*J*_C'Cγ2_ that are dependent on the *χ*^1^ angle of the threonyl side chain (Supplementary Fig. 2 and Supplementary Table 3). Specifically, a large ^3^*J*_NCγ2_ value of ∼1.9 Hz is consistent with a *trans*^*NCγ2*^ relationship between the amide nitrogen and the Cγ2 methyl group of the threonyl head group, corresponding to a *χ*^1^ angle of −60° (*gauche- χ*^1^), whereas a small value of ∼0.2 Hz reflects a *gauche*^*NCγ2*^ relationship (*gauche*+^*NCγ2*^ or *gauche*-^*NCγ2*^), corresponding to *χ*^1^ angles of 180° (*trans χ*^1^) or 60° (*gauche*+ *χ*^1^), respectively. An intermediate value reflects a population-weighted average between the *trans*^*NCγ2*^ and *gauche*^*N*Cγ2^ states^20^. A similar relation is noted for the ^3^*J*_C'Cγ2_ coupling^20^. Thus simultaneous measurements of the ^3^*J*_NCγ2_ and ^3^*J*_C'Cγ2_ scalar couplings enable the determination of the populations of all three rotameric states of the threonyl side chain^19^. Measurements of LPC-011 yielded a ^3^*J*_NCγ2_ coupling of 0.58±0.05 Hz and a ^3^*J*_C'Cγ2_ coupling of 0.77±0.04 Hz, corresponding to a predominant *trans χ*^1^ configuration with a population of 0.65±0.03 (Fig. 1b, Supplementary Table 3). Such an observation is consistent with the ligand conformation in the AaLpxC/LPC-011 crystal structure (Fig. 1a). However, the measurements also revealed that the threonyl side chain of LPC-011 can readily access alternative, minor conformational states with a population of 0.23±0.03 for the *gauche- χ*^1^ conformation and a population of 0.12±0.01 for the *gauche+ χ*^1^ conformation (Fig. 1b). Measurements of CHIR-090 yielded a similar result, with a ^3^*J*_NCγ2_ coupling of 0.45±0.07 Hz and a ^3^*J*_C'Cγ2_ coupling of 0.67±0.04 Hz, corresponding to populations of 0.77±0.04 for the *trans χ*^1^ configuration, 0.14±0.04 for the *gauche- χ*^1^ configuration and 0.09±0.01 for the *gauche+ χ*^1^ configuration (Fig. 1b, Supplementary Table 3). Modelling of the threonyl side chain in the second-most-populated *gauche- χ*^1^ state indicates that the Cγ2 methyl group would experience vdW interactions with the hydrophobic component of the K227 side chain with the Oγ1 hydroxyl group oriented towards solvent, leaving a cavity against the F180 side chain of AaLpxC (Fig. 1c). Although the protein-ligand interactions in the *gauche- χ*^1^ rotameric conformation are less favourable than those in the ground state of the *trans χ*^1^ rotamer, the lack of optimal interactions is partially compensated by the intrinsic free energy difference of the rotameric states of the threonyl side chain that favours the *gauche- χ*^1^ rotamer over the *trans χ*^1^ rotamer in the unbound ligand. Taken together, these solution measurement-derived rotamers collectively portray an inhibitor envelope that can accommodate three substitutions at the Cβ position of the threonyl head group (Fig. 1c).

To test this prediction, we merged the two conformations of the threonyl head group and generated Cβ-di-methyl substituted compounds with the third Cβ-substitution containing either a hydroxyl group (LPC-037) or an amino group (LPC-040) (Supplementary Table 1). Structural analysis of LPC-040 in complex with PaLpxC indeed revealed the anticipated ligand conformation (Fig. 1d; Supplementary Table 2) with the two Cβ-substituted methyl groups forming hydrophobic interactions with F180 (F191^PaLpxC^) and the stem of K227 (K238^PaLpxC^) and the amino group directed towards solvent accessible space to form a water-mediated hydrogen bond with the backbone carbonyl of F180 (F191^PaLpxC^).

We next investigated whether these compounds show enhanced LpxC inhibition in enzymatic assays. *Escherichia coli* LpxC inhibition by CHIR-090 and LPC-011 both displayed slow-binding kinetics consistent with the transition from a rapid-forming initial encounter complex (enzyme-inhibitor complex (EI)) to the stable complex (EI*; Supplementary Fig. 3). Therefore, we focused enzymatic assays on the stable EI* complex. CHIR-090 and LPC-011 are potent LpxC inhibitors with *K*_i_* values of 153±8 pM and 26±1 pM, respectively. Excitingly, the Cβ-triply substituted compounds LPC-037 and LPC-040 both showed enhanced LpxC inhibition, displaying *K*_i_* values of 14±1 pM and 12±1 pM, respectively (Fig. 1e; Supplementary Table 4).

### Drug design from the expanded inhibitor envelope

**Figure 2:**
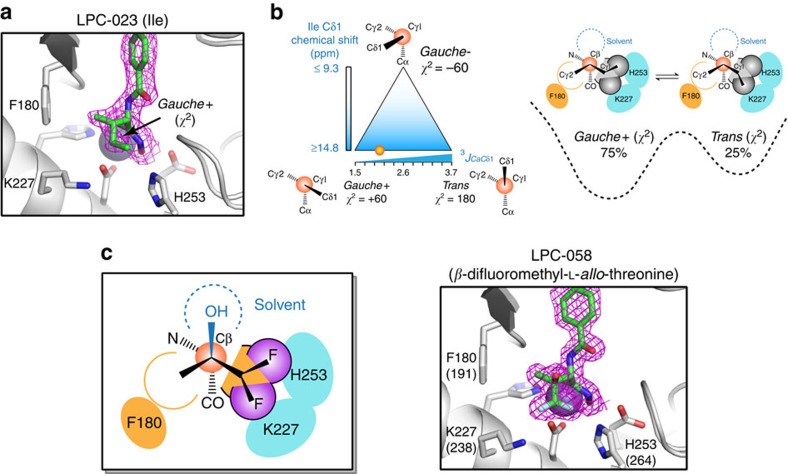
Expanded inhibitor envelope enables the design of a potent inhibitor, LPC-058. (**a**) Crystal structure of AaLpxC in complex with LPC-023, an isoleucine derivative, reveals a *gauche+ χ*^2^ rotamer conformation of the inhibitor. AaLpxC is shown in the cartoon model, with the catalytically important residues in the stick model. LPC-023 is shown in the stick model, with the purple mesh representing the inhibitor omit map (2mFo-DFc) contoured at 1.0σ. (**b**) Combined measurements of the Cδ1 chemical shift and the ^3^*J*_CαCδ1_ coupling of LPC-023 in the protein-bound complex reveal a dynamic equilibrium between *gauche+* and *trans χ*^2^ rotameric states, with the *gauche+* state being the predominant conformation (∼75% population) and the *trans* state being the minor conformation (∼25%). (**c**) Design and structural validation of LPC-058 that optimally occupies the inhibitor envelope. PaLpxC is shown in the cartoon model, with the catalytically important residues in the stick model. Residue numbering reflects the corresponding residues of AaLpxC, with PaLpxC numbers shown in parentheses. LPC-058 is shown in the stick model, with the purple mesh representing the inhibitor omit map (2mFo-DFc) contoured at 1.1σ.

Having delineated the hidden inhibitor envelope at the Cβ position of the threonyl head group, we next examined whether the dynamically accessible envelope of LpxC inhibitors can be further expanded at the γ position. The molecule that fits this purpose is LPC-023 bearing an isoleucine-hydroxamate head group (Supplementary Table 1). Isoleucine shares a basic molecular scaffold with threonine, and its Cγ1-Cδ1 group can be viewed as a substitution of the Oγ1 group of threonine near the conserved lysine (K227^AaLpxC^; K238^PaLpxC^) and histidine (H253^AaLpxC^; H264^PaLpxC^) residues. The isoleucine analogue was crystallized with AaLpxC (Supplementary Table 2), and two copies of the LpxC-inhibitor complexes were found in the asymmetric unit. Among the two protomers of LpxC, the second protomer (chain B) displayed a distorted active site with the catalytic H253 flipped out of the active site in a configuration that has not been observed in any of the previously reported LpxC structures. We reasoned that this would likely reflect a crystallization artifact and consequently focused our analysis on the first LpxC protomer (chain A) in complex with the isoleucine analogue, LPC-023 (Fig. 2a). In this protomer, the isoleucine head group displays a *trans χ*^1^ configuration, consistent with the predominant rotameric state observed in the threonyl group of CHIR-090 and LPC-011. The Cδ1 methyl group adopts a *gauche+ χ*^2^ conformation with regard to the Cα atom. In such a configuration, the Cδ1 methyl group is closest to and potentially forms vdW interactions with the nearby imidazole ring of the catalytic H253. This observation is somewhat surprising as the *gauche+ χ*^2^ angle is rarely observed for isoleucine in protein structures and contributes to <5% of the observed *χ*^2^ rotamers, indicating that such a rotamer represents a high-energy state of the free ligand. We thus investigated whether the Cδ1 methyl group of the isoleucine analogue LPC-023 can access alternative *χ*^2^ rotameric states in solution using the isotopically labelled compound.

The isoleucine Cδ1 chemical shift is sensitive to its *χ*^2^ dihedral angle^21^. For the *gauche+* and *trans χ*^2^ rotamers, isoleucine Cδ1 methyl groups display downfield shifted chemical shifts of >14.8 p.p.m., whereas upfield shifted Cδ1 chemical shifts of <9.3 p.p.m. indicate a *gauche- χ*^2^ conformation. The unbound LPC-023 compound has a Cδ1 chemical shift of 12.8 p.p.m. (Supplementary Fig. 4), consistent with rotameric averaging between a *gauche- χ*^2^ romateric state and the *trans/gauche+* states. In contrast, the LpxC-bound LPC-023 displays a Cδ1 chemical shift of 15.2 p.p.m. (Supplementary Fig. 4), indicating that the *χ*^2^ conformation resides entirely in the *trans* or *gauche+* rotameric states or switches between these two states, but has no detectable population in the *gauche*- state (Fig. 2b, Supplementary Table 5). We next measured the ^3^*J* coupling between the Cδ1 and Cα atoms (Supplementary Fig. 4). A *trans* configuration between Cα and Cδ1 would yield a large scalar coupling of ∼3.7 Hz, whereas a *gauche* configuration would yield a small coupling of ∼1.5 Hz (ref. 21). Our measurements yielded a ^3^*J*_CαCδ1_ coupling of 2.05±0.04 Hz, corresponding to 75±2% population in the *gauche+ χ*^2^ state with the Cδ1 methyl group located adjacent to H253 and 25±2% population in the *trans χ*^2^ state with the same methyl group oriented towards K227 (Fig. 2b, Supplementary Table 5). Although the predominant *gauche+ χ*^2^ rotameric state is consistent with the crystallographically observed inhibitor conformation, our NMR measurements support the notion that both the *gauche+* and *trans* states of the *χ*^2^ rotamers are conformationally populated and they collectively expand the inhibitor envelope at the γ-position, whereas the *gauche- χ*^2^ rotamer is energetically occluded and dynamically inaccessible in solution.

The delineation of two additional pockets that can accommodate methyl-sized functional groups to interact with side chains of the catalytically important histidine and lysine residues suggests fluorine as an attractive functional group for substitution. Fluorine has a slightly smaller size compared with the methyl group^22^, and the fluorine atom is both strongly electronegative and lipophilic^23^. This renders the fluorine group well-suited for forming hydrophobic interactions with the deprotonated histidine side chain and the stem of the lysine group, or forming electrostatic interactions with a protonated histidine imidazolium and a positively charged lysine terminal ammonium group.

Based on this analysis, we introduced difluoro substitution to the pro-*R* methyl group of LPC-037 to yield LPC-058. Structural analysis of LPC-058 with PaLpxC indeed revealed the anticipated ligand conformation (Fig. 2c; Supplementary Table 2), with the β-methyl group occupying the hydrophobic pocket next to F180 (F191^PaLpxC^) for vdW contacts, the β-hydroxyl group residing in the solvent pocket to form a water-mediated hydrogen bond with the backbone of F180 (F191^PaLpxC^), and finally with the difluoromethyl group oriented towards H253 (H264^PaLpxC^) and K227 (K238^PaLpxC^). One of the fluorine atoms adopts a *gauche+* configuration with respect to Cα and forms a hydrogen bond with Nɛ1 atom of the protonated H253 (H264^PaLpxC^), while the second fluorine atom adopts a *trans* configuration with respect to Cα and forms an electrostatic interaction with the ammonium group of K227 (K238^PaLpxC^).

**Figure 3:**
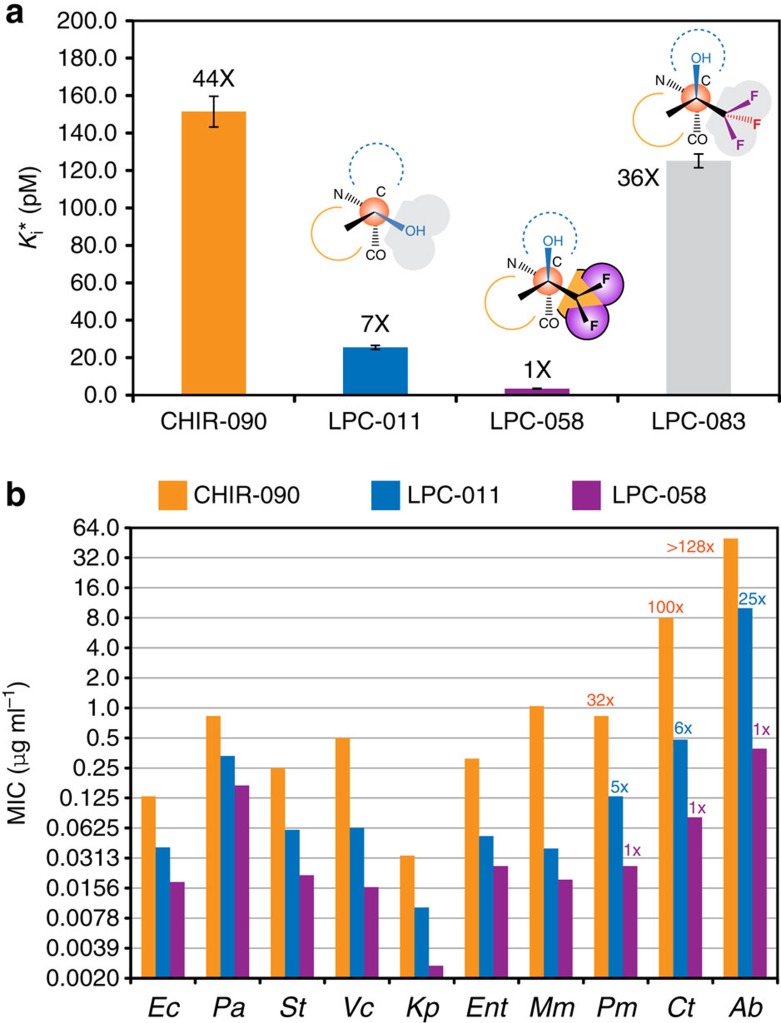
LPC-058 is a superior inhibitor compared with the parent compounds LPC-011 and CHIR-090. (**a**) Inhibition constants of CHIR-090, LPC-011, LPC-058 and LPC-083. The head group of each compound and its conformation within the inhibitor envelope is denoted. (**b**) LPC-058 is a potent antibiotic and displays enhanced activity over LPC-011 and CHIR-090 against a diverse array of Gram-negative pathogens. MIC enhancement of >4-fold over LPC-011 and ≥32-fold over CHIR-090 is labelled. Tested bacterial species include *E. coli* (Ec), *P. aeruginosa* (Pa), *Salmonella typhimurium* (St), *Vibrio cholerae* (Vc), *Klebsiella pneumoniae* (Kp), *Enterobacter cloacae* (Ent), *Morganella morganii* (Mm), *Proteus mirabilis* (Pm), *Chlamydia trachomatis* (Ct) and *Acinetobacter baumannii* (Ab).

Excitingly, LPC-058 is an exceptionally potent inhibitor. It displayed slow-binding kinetics consistent with the rapid formation of an initial encounter complex (EI) followed by slow transition to the stable EI* complex (Supplementary Fig. 5). Accordingly, *k*_obs_ increased hyperbolically over the inhibitor concentration^24^. Steady-state kinetics analysis of the stable EI* complex revealed an inhibition constant (*K*_i_*) of 3.5±0.2 pM, a 7-fold enhancement of potency over LPC-011 and a 44-fold enhancement over CHIR-090 (Fig. 3a). Incorporation of the *K*_i_* value into the analysis of the inhibitor concentration-dependent *k*_obs_ values enabled accurate determination of the forward rate (*k*_5_=0.39±0.02 min^−1^) and reverse rate (*k*_6_=0.0014±0.0001, min^−1^) from EI to EI* and the inhibition constant of the initial encounter complex EI (*K*_i_=973±128 pM).

To examine whether LPC-058 designed from the cryptic inhibitor envelope shows improved antibiotic activity over CHIR-090 and LPC-011, we determined its minimum inhibitory concentration (MIC) values against a range of Gram-negative pathogens. LPC-058 showed uniform improvement over CHIR-090 and LPC-011 against all Gram-negative bacterial strains tested (Fig. 3b, Supplementary Table 6). In general, enhanced antibiotic activities of 2- to 4-fold over LPC-011 and 5- to 55-fold over CHIR-090 were observed for *E. coli, P. aeruginosa*, *Salmonella typhimurium*, *Vibrio cholerae*, *Klebsiella pneumoniae*, *Enterobacter cloacae* and *Morganella morganii.* More pronounced improvements (5- to 25-fold over LPC-011 and 32- to >128-folds over CHIR-090) were observed for *Proteus mirabilis*, *Chlamydia trachomatis* and *Acinetobacter baumannii*. Of particular importance is the potent antibiotic activity of LPC-058 against *Acinetobacter baumannii* (MIC=0.39 μg ml^−1^). To the best of our knowledge, LPC-058 is the first reported LpxC inhibitor with an MIC value below 1 μg ml^−1^ against this clinically important Gram-negative pathogen *in vitro*. The broad-spectrum antibiotic activity of LPC-058 highlights the therapeutic potential of LpxC inhibitors as effective antibiotics against a wide range of Gram-negative infections.

## Discussion

It is widely acknowledged that the dynamic interconversion of multiple conformational states is an intrinsic property of proteins and nucleic acids in solution. In comparison, the conformational dynamics of small molecules in their receptor-bound states has rarely been investigated, let alone utilized for drug design. Here we show that small-molecule inhibitors of LpxC dynamically access alternative, minor-state ligand conformations in addition to the predominant conformational state observed in crystal structures. These minor-state ligand conformations, together with that of the major state, collectively delineate a cryptic inhibitor envelope in solution that is invisible to crystallographic studies. Furthermore, we show that such a cryptic inhibitor envelope provides important molecular insights for the design of high-affinity ligands. In the case of LpxC inhibitors, analysis of the inhibitor envelope has led to the development of a potent antibiotic LPC-058. With inclusion of only three additional heavy atoms, the newly designed compound LPC-058 enhanced the inhibitory effect towards *E. coli* LpxC over its parent compound LPC-011 by 7-fold and improved antibiotic activity by 2- to 25-fold against a wide range of Gram-negative pathogens, rendering it the most potent and the most broad-spectrum LpxC inhibitor *in vitro*. Although some features of the LpxC inhibitor envelope, such as the solvent accessible pocket at the Cβ position of the threonyl head group, might have been envisaged based on structural analysis of the LpxC/CHIR-090 complex^14^ and LpxC inhibitors bearing similar head groups to LPC-037 and LPC-040, but different tail groups, have been reported^25^,^26^, the precise definition of two accessory pockets at the Oγ1-position of the threonyl head group could not have been predicted by structural analysis alone. In fact, the most widely employed functional substitution of a pro-*R* methyl of LPC-037 is the trifluoromethyl group (CF_3_), not the difluoromethyl group (CF_2_) utilized in LPC-058. However, the trifluoromethyl substituted compound LPC-083 compromised the inhibitory effect (*K*_i_*=125±4 pM) over its parent compound LPC-037 (*K*_i_*=14±1 pM) by nearly ninefold (Supplementary Table 4). Its inhibition constant is worse than LPC-011 by fivefold (Fig. 3a), and it is a less potent antibiotic against *E. coli* (MIC=0.1 μg ml^−1^) than LPC-011 (MIC=0.04 μg ml^−1^), which would have argued away from development of the synthetically more challenging β-difluoromethyl-*allo*-threonyl compound LPC-058 designed from the dynamic inhibitor envelope.

The work presented here departs from the established paradigm of ligand design from the crystallographically visible, static ligand conformation and highlights the potential of drug development from the ‘invisible', dynamically accessible inhibitor envelope in solution, which encompasses the receptor-bound ligand conformations from both major and minor states. The framework presented here should be broadly applicable to lead optimization campaigns for small molecules, peptides and peptidomimetics to yield more effective therapeutics.

## Methods

### Chemical synthesis

Details of chemical synthesis and characterization are described in Supplementary Methods.

### Crystallography structural analysis

Protein samples of AaLpxC and PaLpxC were prepared as described previously^16^. Before crystallization trials, a fourfold molar excess of each compound, dissolved in DMSO, was mixed with 8 mg ml^−1^ AaLpxC (1-275, C181A) in 100 mM potassium chloride, 2 mM dithiothreitol  and 25 mM HEPES (pH 7.0) or 12 mg ml^−1^ PaLpxC (1-299, C40S) in 50 mM sodium chloride, 2 mM tris(2-carboxyethyl)phosphine and 25 mM HEPES (pH 7.0), respectively. For PaLpxC, 10 mM zinc sulfate was added as a crystallization additive. The protein-inhibitor mixture was incubated for 30 min at room temperature to obtain a homogenous sample. All of the LpxC-inhibitor complex crystals were obtained by the sitting-drop vapour diffusion method at 20 °C. Initial crystallization screening yielded microcrystals for the AaLpxC/LPC-011 complex in a reservoir solution containing 0.1 M HEPES (pH 7.0) and 15% PEG 8000 and for the AaLpxC/LPC-023 complex in a reservoir solution containing 0.18 M ammonium chloride, 11.8% PEG3350 and 4% 1,3-propanediol. The microcrystals were used to prepare seeding stocks by the Seed-Bead protocol (Hampton Research, HR2-320). Diffraction quality crystals were obtained by the streak-seeding method. The final crystallization reservoirs contained 0.05 M ammonium acetate and 10% PEG3350 for the AaLpxC/LPC-011 complex and 0.18 M ammonium chloride, 11.8% PEG3350 and 10% 1,3-propanediol for the AaLpxC/LPC-023 complex, respectively. High quality crystals of the PaLpxC/LPC-040 and PaLpxC/LPC-058 complexes were obtained in precipitant solutions containing 0.1 M sodium acetate trihydrate (pH 4.8–5.1) and 2.4–2.6 M ammonium nitrate. Crystals were cryoprotected using the corresponding mother liquor solutions containing 30% 2-methyl-2,4-pentanediol (MPD) for the AaLpxC/LPC-011 complex, 30% ethylene glycol for the AaLpxC/LPC-023 complex and 10% glycerol for the PaLpxC/inhibitor complexes, respectively, before flash-freezing for data collection.

Data sets of the PaLpxC/LPC-040 and PaLpxC/LPC-058 complexes were collected in-house using a Rigaku MicroMax-007 HF rotating anode generator and R-Axis IV++ detector. Data sets of the AaLpxC/LPC-011 and AaLpxC/LPC-023 were collected at the SER-CAT 22-ID beamline at the Advanced Photon Source at Argonne National Laboratory. The collected X-ray diffraction data were processed using HKL2000 (ref. 27) or XDS (ref. 28). The crystal structures of LpxC-inhibitor complexes were solved by molecular replacement with the programme PHASER (ref. 29) using PDB entries 3P3C and 3P3E for the AaLpxC-inhibitor complexes and the PaLpxC-inhibitor complexes, respectively. Restraints of the inhibitors were generated by using eLBOW (ref. 30) and edited manually. Iterative model building and refinement was carried out using COOT (ref. 31) and PHENIX (ref. 32). The 2mFo-DFc omit maps^33^ were generated using PHENIX^32^.

### Solution NMR measurements

Deuterated AaLpxC was expressed and purified as described previously^13^. The AaLpxC-inhibitor complexes were prepared by adding individual inhibitors to the purified protein in the presence of 5% deuterated dimethylsulfoxide (DMSO) in a 1:2 protein-inhibitor molar ratio, and incubated initially at room temperature and then at 45 °C to form the complex. Samples were concentrated and exchanged into the NMR buffer containing 25 mM sodium phosphate pH 7.0, 100 mM KCl, 5% deuterated DMSO and 100% D_2_O. The NMR sample concentration was ∼1 mM.

The scalar couplings of ^3^*J*_C'Cγ2_ and ^3^*J*_NCγ2_ for the AaLpxC/CHIR-090 and AaLpxC/LPC-011 complexes were measured on a Bruker 700 MHz NMR spectrometer at 45 °C, using J-modulated ^1^H–^13^C constant-time HSQC experiments^34^,^35^. The reference and scalar coupling-modulated CT-HSQC spectra were recorded in an interleaved manner with a constant-time delay (2T) set to 57.4 ms, and the maximum evolution time for the indirect (^13^C) dimension set 12.1 ms. Data were processed using NMRPIPE (ref. 36) with eightfold zero-filling in the indirect dimension. The peak intensities were measured by SPARKY (ref. 37), and the ^3^*J*_C'Cγ2_ and ^3^*J*_NCγ2_ couplings were calculated from the ratio of the peak intensities between the reference spectrum (I_ref_) and the J-modulated spectrum (I_mod_) according to equation (1):


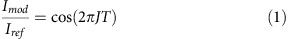


Rotameric populations were calculated based on the three-site jump model^19^ using values derived from self-consistent parameterization of ^3^*J* couplings^20^.

The scalar coupling ^3^*J*_CαCδ1_ for the AaLpxC/LPC-023 complex was measured on an Agilent 800 MHz NMR spectrometer at 37 °C using a J-modulated constant-time ^13^C HSQC experiment using selective Ile-Cα inversion pulses. The ^3^*J*_CαCδ1_ coupling was calculated from the ratio of the peak intensities between the reference spectrum (I_ref_) and the J-modulated spectrum (I_mod_) according to equation (1). Since the Cδ1 chemical shift of 15.2 p.p.m. of LPC-023 excludes the *gauche- χ*^2^ rotamer^21^, populations of the remaining rotamers were calculated from ^3^*J*_CαCδ1_ based on the two-site jump model between the *gauche+* and *trans* rotameric states^21^.

### Enzymatic assays

The radiolabelled substrate for the LpxC enzymatic assays, [α-^32^P] UDP-3-*O*-[(*R*)-3-hydroxymyristoyl]-*N*-acetylglucosamine, and the unlabelled carrier substrate were prepared as previously described^38^. The assays were performed in a buffer consisting of 25 mM HEPES pH 7.4, 100 mM KCl, 1 mg ml^−1^ BSA, 2 mM dithiothreitol and 5 μM substrate at 30 °C. Serial twofold dilutions of each inhibitor were prepared in DMSO and added to the reaction mixture with a 10-fold dilution. The assays were initiated by addition of purified LpxC protein into the reaction mix with 1:4 dilution to the final concentration as specified.

The *K*_M_ value was determined by varying substrate concentrations from 0.4 to 50 μM with 0.2 nM of LpxC. To study the slow, tight-binding inhibition, LpxC activity was assessed in the presence of varying inhibitor concentrations. The product conversions were determined from 15 s up to 2 h after addition of 0.2 nM enzyme for CHIR-090 and LPC-011 in the presence of 5 μM substrate. Time-dependent inhibition of LPC-058 was assayed in the presence of 30 μM substrate and 0.1 nM enzyme such that *k*_obs_ can be extracted under the slow, but not tight-binding conditions^39^. The following time-dependent equation was used to fit the data:


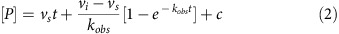


with *v*_*s*_ representing the steady-state rate, *v*_*i*_ the initial rate, *k*_obs_ the rate of transition from the initial encounter complex to the final complex and *c* the baseline.

The *K*_i_* was determined by analysing the rate of product accumulation after formation of the stable EI* complex. IC_50_ curves for individual compounds were determined in the presence of 20 pM of the enzyme and varying inhibitor concentrations. The Morrison's quadratic equation was used to fit the fractional activity data to determine *K*_i_^*app^:


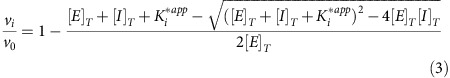


where [E]_T_ and [I]_T_ represent the total enzyme and inhibitor concentrations, respectively. The inhibition constant *K*_i_* is converted from *K*_i_^*app^ according to the following relationship:


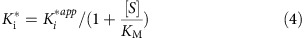


For two-step slow-binding inhibition, kinetic parameters *k*_5_, *k*_6_ and *K*_i_ were extracted from curve fitting of experimental *k*_obs_ values to inhibitor concentrations based on equations (5 and 6).


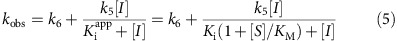



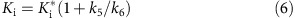


### Measurements of the minimum inhibitory concentration (MIC)

The MIC assay protocol was adapted from methods described in National Committee for Clinical Laboratory Standards (NCCLS) to using 96-well plates^40^. Bacteria were grown in the Mueller–Hinton medium at 37 °C in the presence of varying concentrations of inhibitors and 5% DMSO. To obtain more accurate readings of the MICs, three series of twofold dilutions of inhibitors were used. The starting concentrations of the three series are different by factors of 1.33 and 1.67, respectively. MICs were reported as the lowest compound concentration that inhibited bacterial growth.

## Additional information

**Accession codes:** The coordinates for the X-ray structures have been deposited to the Protein Data Bank (PDB) with accession codes of 5DRO, 5DRQ, 5DRP and 5DRR for the AaLpxC/LPC-011, PaLpxC/LPC-040, AaLpxC/LPC-023 and PaLpxC/LPC-058 complexes, respectively.

**How to cite this article:** Lee, C.-J. *et al*. Drug design from the cryptic inhibitor envelope. *Nat. Commun.* 7:10638 doi: 10.1038/ncomms10638 (2016).

## Supplementary Material

Supplementary Information Supplementary Figures 1-5, Supplementary Tables 1-6, Supplementary Methods and Supplementary References
