# Supplementary material for: Drug design from the cryptic inhibitor envelope
Source: Nat Commun. 2016 Feb 25;7:10638. doi: 10.1038/ncomms10638 (PMC4773385; doi:10.1038/ncomms10638)
Supplement: Supplementary Information — Supplementary Figures 1-5, Supplementary Tables 1-6, Supplementary Methods and Supplementary References [file ncomms10638-s1.pdf]

Supplementary Figures and Tables

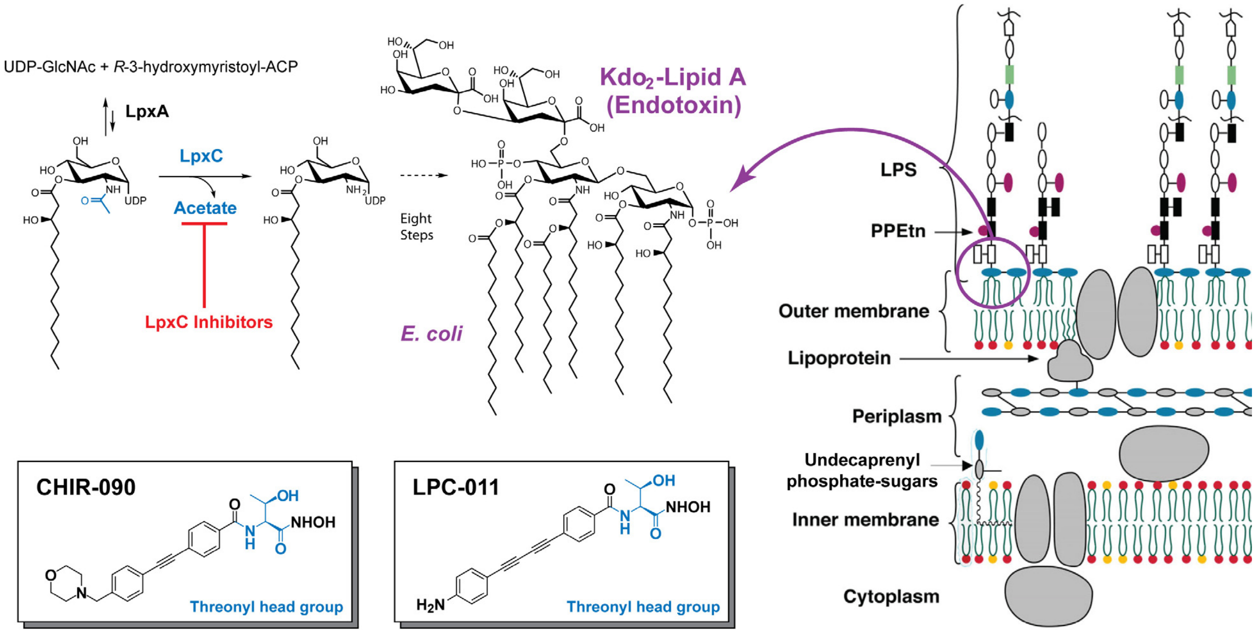

Supplementary Figure 1 | LpxC-targeting antibiotics disrupt the biosynthesis of lipid A. Lipid A is the hydrophobic anchor of lipopolysaccharide (LPS) and the predominant lipid species in the outer leaflet of the outer membrane of Gram-negative bacteria.

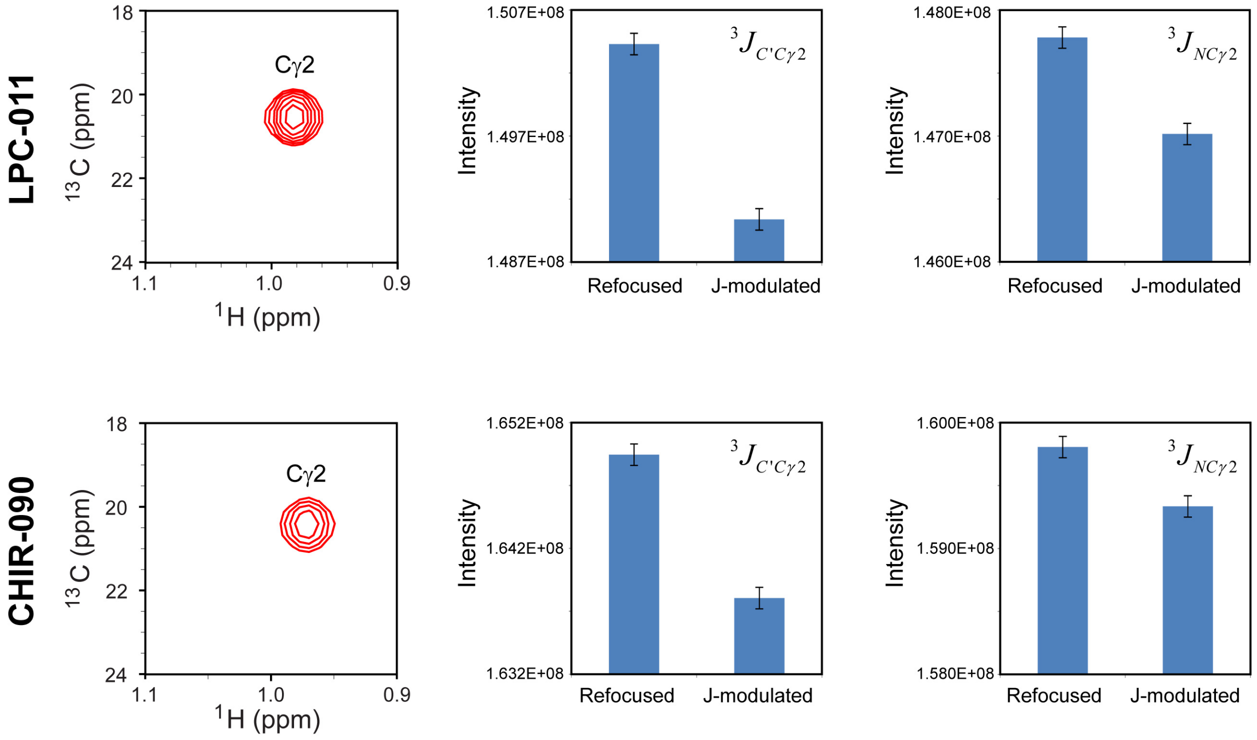

Supplementary Figure 2 | Scalar coupling measurements of LPC-011 and CHIR-090 bound to LpxC. NMR spectra of the Cγ2 methyl signal of LPC-011 and CHIR-090 are shown in the left panels. Intensity measurements of the refocused and J-modulated experiments for $^{3}J_{C'C\gamma2}$ and $^{3}J_{NC\gamma2}$ are shown in the middle and right panels, respectively.

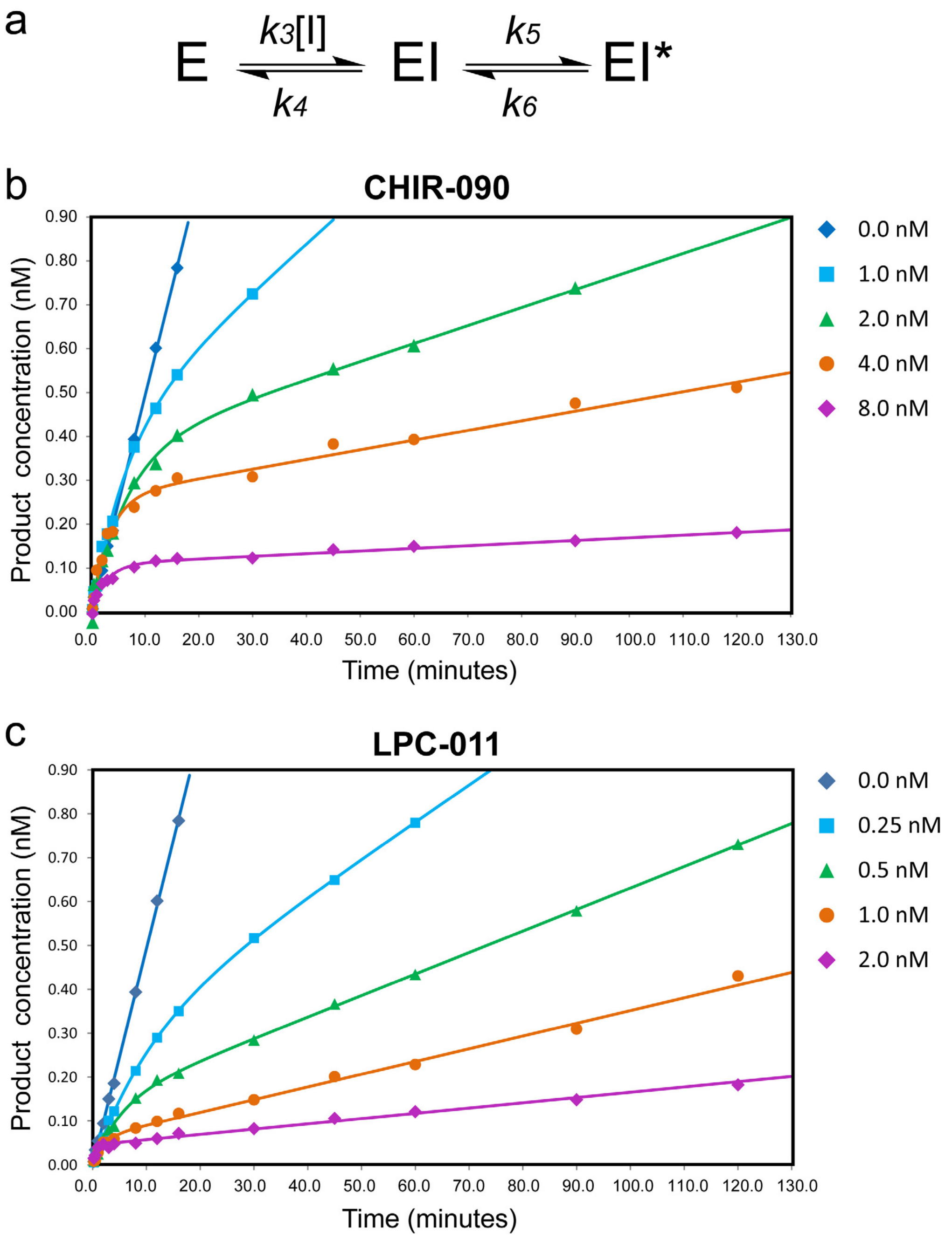

Supplementary Figure 3 | Time-dependent inhibition of EcLpxC by CHIR-090 and LPC-011. (**a**) Two-step slow-binding kinetics of LpxC inhibitors. Panels (**b)** and (**c)** show nonlinear LpxC product accumulation in the presence of varying concentrations of CHIR-090 and LPC-011, respectively, reflecting the transition from the initial enzyme-inhibitor encounter complex (EI) to the stable complex (EI*).

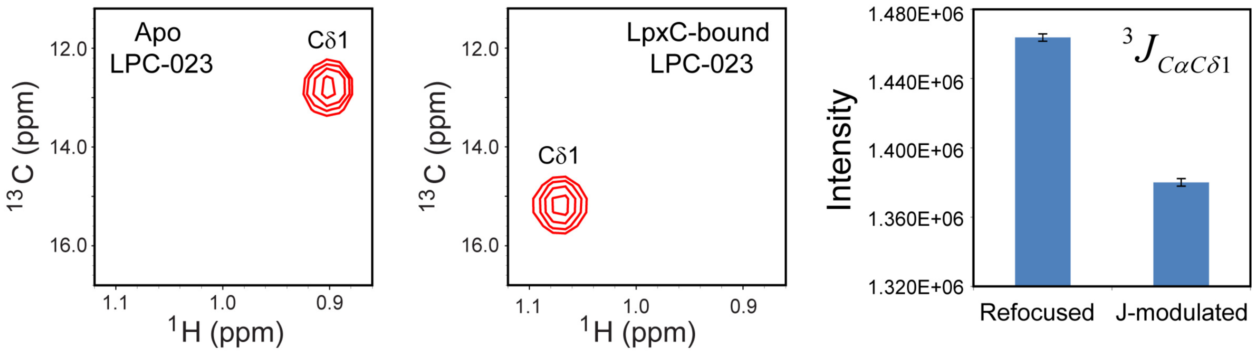

Supplementary Figure 4 | NMR measurements of LPC-023. NMR spectra of the Cδ1 methyl group of LPC-023 in the apo and LpxC-bound states are shown in the left and middle panels, respectively. Intensity measurements of the refocused and J-modulated experiments for $^{3}J_{C\alpha C\delta 1}$ are shown in the right panel.

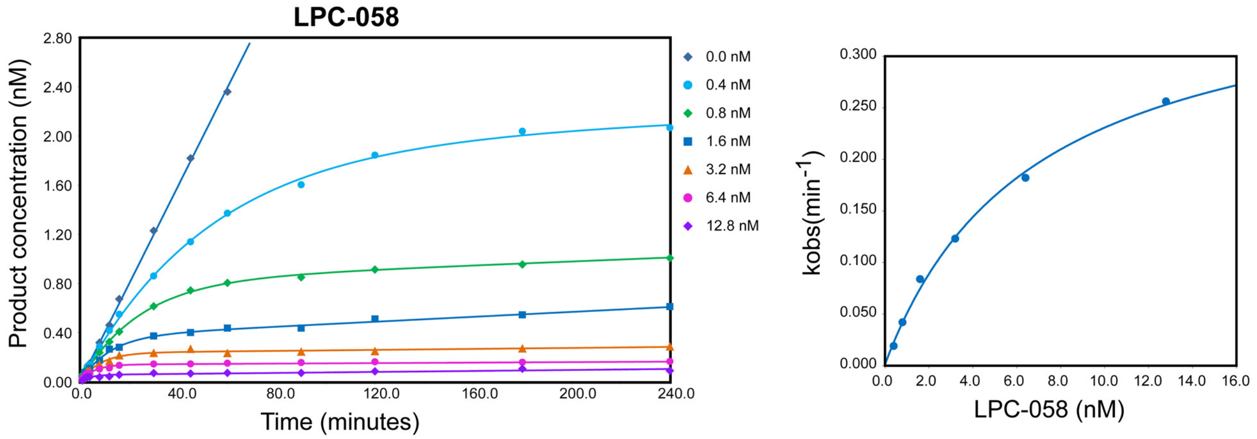

Supplementary Figure 5 | Time-dependent inhibition of EcLpxC by LPC-058. EcLpxC displays nonlinear product accumulation in the presence of varying concentrations of LPC-058. A hyperbolic increase of k_{obs} over inhibitor concentration was observed, consistent with the transition from the initial enzyme-inhibitor encounter complex (EI) to the stable complex (EI*). Under slow, not tight binding condition, the k_{obs} values derived from local fitting of the product accumulation curve were fit using Eqs. [5] and [6] with a constrained K_i^* value of 3.5 ± 0.2 pM. The fitting yielded the following parameter values: K_i = 973 ± 128 pM, k_5 = 0.39 ± 0.02 min^{-1}, and k_6 = 0.0014 ± 0.0001 min^{-1} (R^2=0.996).

Supplementary Table 1: Chemical structures of LpxC inhibitors

Name	Structure	Name	Structure
**CHIR-090 (Thr)**	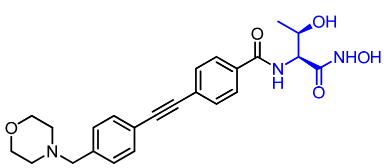	**LPC-011 (Thr)**	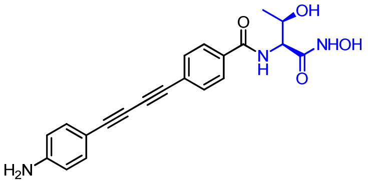
**LPC-037 (β-hydroxy-Val)**	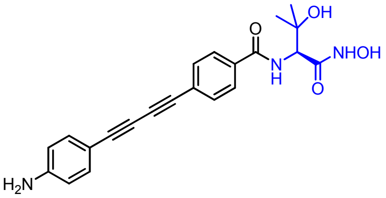	**LPC-040 (β-amino-Val)**	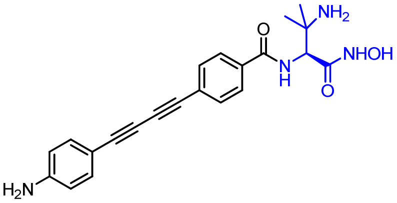
**LPC-023 (Ile)**	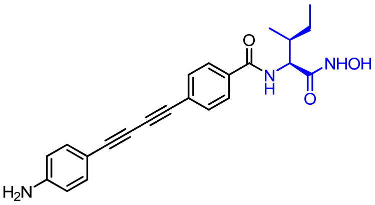	**LPC-058**	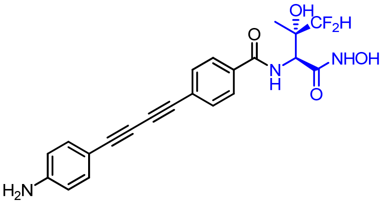
**LPC-083**	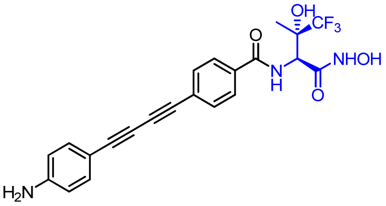		

Supplementary Table 2: Data collection and refinement statistics

	AaLpxC/ LPC-011	PaLpxC/ LPC-040	AaLpxC/ LPC-023	PaLpxC/ LPC-058
**Data collection**				
Space group	P 21 21 21	P 21 21 21	P 1	P 21 21 21
Cell dimensions				
a, *b*, *c* (Å)	54.8, 74.8, 135.9	52.4, 74.0, 88.6	45.7 50.4 61.7	52.8, 73.9, 88.2
α, β, γ (°)	90, 90, 90	90, 90, 90	80.3, 71.7, 88.9	90, 90, 90
Resolution (Å)	67.94 - 2.01 (2.08 - 2.01)	50.00 - 1.63 (1.66 - 1.63)	50.00 - 1.84 (1.87 - 1.84)	28.32 - 1.59 (1.68 - 1.59)
R_{sym} or R_{merge}	11.9 (51.3)	5.4 (40.3)	5.3 (31.1)	4.7 (42.4)
$I / \sigma I$	21.67 (5.05)	16.56 (4.25)	24.5 (3.2)	15.01 (2.45)
Completeness (%)	97.2 (92.1)	99.7 (99.6)	97.7 (96.5)	97.7 (94.5)
Redundancy	14.4 (11.1)	6.3 (6.6)	4.4 (4.4)	5.0 (4.8)
**Refinement**				
Resolution (Å)	67.94 - 2.01 (2.08 - 2.01)	17.87 - 1.63 (1.69 - 1.63)	34.81 - 1.89 (1.96 - 1.89)	28.32 - 1.59 (1.65 - 1.59)
Unique reflections	36885 (3708)	43373 (4171)	40342 (3995)	46039 (4405)
Redundancy	14.4 (11.1)	6.3 (6.6)	4.4 (4.4)	5.0 (4.8)
R_{work} / R_{free}	0.156/0.186	0.170/0.203	0.156/0.192	0.179/0.210
No. atoms	4840	2742	4619	2690
Protein	4370	2332	4296	2328
Ligand/ion	70	147	64	101
Water	400	263	259	261
Average *B*-factors	21.90	26.60	34.90	26.50
Protein	20.80	24.80	34.50	25.50
Ligand/ion	29.30	37.20	37.30	31.80
Water	31.90	36.60	40.90	33.70
R.m.s. deviations				
Bond lengths (Å)	0.002	0.009	0.006	0.004
Bond angles (°)	0.69	1.16	0.99	0.80
Ramachandran				
Favored (%)	96.8	97.4	97.0	97.7
Outliers (%)	0.0	0.0	0.0	0

*Values in parentheses are for highest-resolution shell.

Supplementary Table 3: Scalar couplings and rotameric populations of CHIR-090 and LPC-011

Compounds	$^{3}J_{C'C\gamma2}$ (Hz)	$^{3}J_{NC\gamma2}$ (Hz)	χ^1 P_{trans}	χ^1 $P_{gauche-}$	χ^1 $P_{gauche+}$
**CHIR-090**	0.67 ± 0.04	0.45 ± 0.07	0.77 ± 0.04	0.14 ± 0.04	0.09 ± 0.01
**LPC-011**	0.77 ± 0.04	0.58 ± 0.05	0.65 ± 0.03	0.23 ± 0.03	0.12 ± 0.01

Supplementary Table 4: K_i^* values of LpxC inhibitors

Compounds	CHIR-090	LPC-011	LPC-037	LPC-040	LPC-058	LPC-083
K_i^* (pM)	152 ± 8	26 ± 1	14 ± 1	12 ± 1	3.5 ± 0.2	125 ± 4

Supplementary Table 5: Chemical shift, scalar coupling, and rotameric populations of LPC-023

Cδ1 Shift (ppm)	$^{3}J_{C\alpha C\delta1}$ (Hz)	χ^2 P_{trans}	χ^2 $P_{gauche+}$
15.2	2.05 ± 0.04	0.25 ± 0.02	0.75 ± 0.02

Supplementary Table 6: MIC values of LpxC inhibitors

**Bacterial Strains**	**CHIR-090 MIC (µg/mL)**	**LPC-011 MIC (µg/mL)**	**LPC-058 MIC (µg/mL)**	**Enhancement ($MIC_{LPC-011}$/ $MIC_{LPC-058}$)**	**Enhancement ($MIC_{CHIR-090}$/ $MIC_{LPC-058}$)**
Escherichia coli **W3110**	0.13	0.04	0.018	2.2	7.2
Pseudomonas aeruginosa **PAO1**	0.83	0.33	0.167	2.0	5.0
Salmonella typhimurium **LT2**	0.25	0.06	0.021	2.8	12
Vibrio cholerae **P4 (P27459DctxAB::KmR, SmR)**	0.50	0.063	0.016	3.9	31
Klebsiella pneumoniae **10031**	0.033	0.01	0.0026	3.8	13
Enterobacter cloacae	0.31	0.052	0.026	2.0	12
Morganella morganii	1.04	0.039	0.019	2.0	55
Proteus mirabilis	0.83	0.13	0.026	5.0	32
Chlamydia trachomatis *	8	0.48	0.08	6.0	100
Acinetobacter baumannii **17978**	>50	10	0.39	25	>128

*Minimal chlamydiacidal concentration (MCC)

Supplementary Methods

Chemical Synthesis

The synthesis of CHIR-090, isotope labeled CHIR-090, and LPC-011 was reported previously [1, 2]. Synthesis of the remaining compounds is outlined below.

Synthesis of LPC-023:

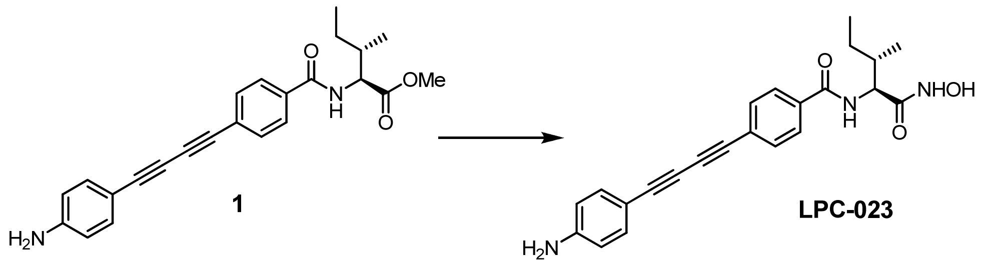

Compound **1**: Methyl (2S, 3R)-2-({4'-[4'-(4'-aminophenyl)buta-1',3'-diyn-1'-yl]phenyl} formamido)-3'-methylpentanoate. To a stirred mixture of 4-((4-aminophenyl)buta-1,3-diyn-1-yl)benzoic acid [3] (100 mg, 0.38 mmol) and L-Isoleucine methyl ester hydrochloride (98 mg, 0.54 mmol, 1.40 equiv) in anhydrous DMF (5 mL) was added N-ethyl-N'-(3-dimethylaminopropyl) carbodiimide hydrochloride (103 mg 0.54 mmol, 1.40 equiv), 1-hydroxybenzotriazole (73 mg, 0.54 mmol, 1.40 equiv) at room temperature. The mixture was cooled with an ice-bath, and diisopropylethylamine (0.27 mL, 1.53 mmol, 4 equiv) was added. The reaction mixture was stirred under argon and at 0°C for 1h, and then was allowed to warm to temperature with the stirring continued for additional 14h. The resulting yellow solution was condensed to dryness with a rotary evaporator, and the residue was treated with water (20 mL) and extracted with EtOAc (3×30 mL). The combined extracts were washed with brine (20 mL), and dried over anhydrous Na_2SO_4. Evaporation of the solvent afforded the crude products, which was purified by flash chromatography (eluting with 1-2% MeOH in DCM) to afford **1** (134 mg, 91% yield) as yellow solid. NMR: ^1H (300 MHz, DMSO-d_6) δ (ppm) 0.82-0.89 (m, 6H), 1.19-1.27 (m, 1H), 1.46-1.49 (m, 1H), 1.92-1.95 (m, 1H), 3.63 (s, 3H), 4.32 (t, *J*=15.3 Hz, 1H), 5.84 (s, 2H), 6.53 (d, *J*=8.7 Hz, 2H), 7.24 (d, *J*=8.7 Hz, 2H), 7.63 (d, *J*=8.4 Hz, 2H), 7.88 (d, *J*=8.4 Hz, 2H), 8.72 (d, *J*=7.5 Hz, 1H); ^{13}C (75 MHz, DMSO-d_6) δ (ppm) 11.55, 16.19, 25.91, 36.28, 52.34, 58.12, 71.77, 77.35, 80.82, 86.40, 105.80, 114.26, 124.87, 128.74, 132.59, 134.71, 151.57, 166.73, 172.87; LC-MS: $[M+H]^+$ 389.

Compound **LPC-023**: (2S,3R)-2-({4'-[4'-(4'-aminophenyl)buta-1',3'-diyn-1-yl]phenyl} formamido)-N-hydroxy-3-methylpentanamide. To an ice-cold solution of **1** (100 mg, 0.26 mmol) dissolved in anhydrous MeOH (1 mL) and THF (1 mL) was added hydroxylamine hydrochloride (93 mg, 1.34 mmol, 5.0 equiv) followed by 25% sodium methoxide in methanol solution (0.49 mL, 2.03 mmol, 7.5 equiv). The reaction mixture was stirred under argon and at 0°C for 2h, and then was allowed to warm to ambient temperature with the stirring continued overnight (14h). The resulting yellow suspension was condensed to dryness with a rotary evaporator, and the residue was treated with water (30 mL), extracted with EtOAc (3×30 mL). The combined extracts were washed with brine (20 mL), and dried over anhydrous Na_2SO_4. Evaporation of the

solvent afforded the crude product, which was purified by CombiFlash silica gel chromatography (eluting with 2-5% MeOH in DCM) to afford the product (60 mg, 60% yield) as yellow solid. NMR: ^{1}H (300 MHz, DMSO-d_6) δ (ppm) 0.78-0.85 (m, 6H), 1.12-1.16 (m, 1H), 1.46-1.53 (m, 1H), 1.88-1.93 (m, 1H), 4.14 (t, *J*=18.3 Hz, 1H), 5.84 (s 2H), 6.53 (d, *J*=8.7 Hz, 2H), 7.24 (d, *J*=8.4 Hz, 2H), 7.60 (d, *J*=8.4 Hz, 2H), 7.88 (d, *J*=8.4 Hz, 2H), 8.55 (d, *J*=8.7 Hz, 1H), 8.88 (s, 1H), 10.74 (s, 1H); ^{13}C (75 MHz, DMSO-d_6) δ (ppm) 11.17, 16.00, 25.56, 35.97, 56.13, 71.80, 77.22, 80.86, 86.32, 105.82, 114.26, 124.63, 128.64, 132.54, 134.70, 135.04, 151.55, 166.03, 168.36; HRMS: calculated for $C_{23}H_{23}N_3O_3$ 389.1739; found 389.1731 M^+.

Synthesis of LPC-037

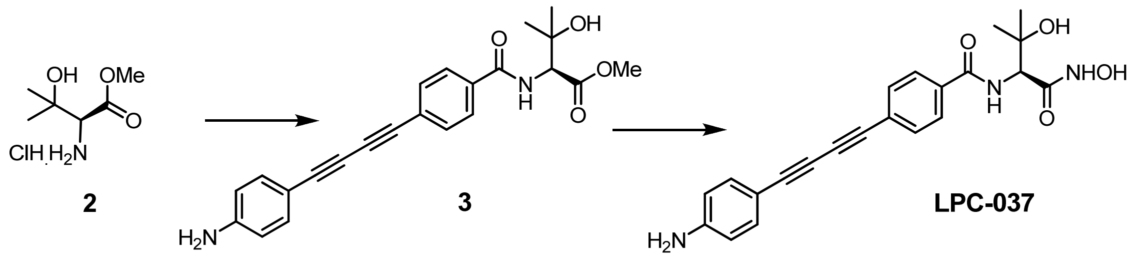

Compound **2**: Methyl (S)-(+)-2-amino-3-hydroxy-3-methylbutanoate. To an ice-cold anhydrous MeOH (8 mL) was added dropwise thionyl chloride (0.54 mL, 7.51 mmol, 2.0 equiv), and the solution was stirred at rt for 5 min. (S)-(+)-2-amino-3-methylbutanoic acid (500 mg, 3.76 mmol) was then added to the solution in one portion, and then the reaction mixture was heated at 70°C for 2h. LC-MS analysis showed the completion of the reaction. After the resulting mixture was cooled to ambient temperature, the solvents were evaporated to dryness and then pumped under high vacuum for 4h, obtaining the crude **2** (619 mg, 90% yield) as white solid. NMR: ^{1}H (300 MHz, CD_3OD) δ (ppm) 1.25 (s, 3H), 1.44 (s, 3H), 3.85 (s, 3H), 3.92 (s, 1H); ^{13}C (75 MHz, CD_3OD) δ (ppm) 23.71, 27.43, 52.50, 61.81, 69.19, 167.97; LC-MS: $[M+H]^+$ 148.2.

Compound **3**: (S)-Methyl 2-(4-((4-aminophenyl)buta-1,3-diynyl)benzamido)-3-hydroxy-3-methylbutanoate. To a stirred mixture of compound 4-((4-aminophenyl)buta-1,3-diyn-1-yl)benzoic acid [3] (200 mg, 0.76 mmol) and compound **2** (300 mg, 0.91mmol, 1.20 equiv) in anhydrous DMF (5 mL) was added EDC.HCl (204 mg 1.07 mmol, 1.20 equiv), HOBt (145mg, 1.87 mmol, 1.20 equiv) at rt. The reaction mixture was cooled with an ice-bath, and diisopropylethylamine (1.06 mL, 7.12 mmol, 8.0 equiv) was added. The reaction mixture was stirred under argon at 0°C for 1h, and then was allowed to warm to ambient temperature with the stirring continued for additional 14h. The resulting yellow solution was condensed to dryness with a rotary evaporator, and the residue was treated with water (50 mL). The mixture was extracted with EtOAc (3×50 mL). The combined extracts were washed with water (30 mL), brine (30 mL) and dried over anhydrous Na_2SO_4. Evaporation of the solvent was afforded the crude product, which was purified by flash chromatography (eluting with 0-6% MeOH in DCM) to afford **6** (240 mg, 80% yield) as red solid. NMR: ^{1}H (300 MHz, CD_3OD) δ (ppm) 1.32 (s, 3H), 1.36 (s, 3H), 3.76 (s, 3H), 4.64 (s, 1H), 6.61 (d, *J*=8.7 Hz, 2H), 7.24 (d, *J*=8.7 Hz, 2H), 7.58 (d, *J*=8.7 Hz, 2H), 7.83 (d, *J*=8.7 Hz, 2H); ^{13}C (75 MHz, CD_3OD) δ (ppm) 26.11, 26.41, 51.48, 61.60, 70.83, 71.38, 76.78, 79.01, 84.67, 108.30, 114.24, 126.14, 127.54, 132.16, 133.86, 150.21, 168.08, 177.11; LC-MS: $[M+H]^+$391.

Compound **LPC-037**: (2S)-2-({4'-[4'-(4'-aminophenyl)buta-1',3'-diyn-1-yl]phenyl} formamido) -N,3'-dihydroxy-3'-methylbutanamide. To an ice-cold solution of compound **3** (120 mg, 0.31 mmol) dissolved in anhydrous MeOH (1 mL) and THF (1 mL) was added hydroxylamine hydrochloride (107 mg, 1.54 mmol 5.0 equiv), followed by 25% sodium methoxide in methanol solution (0.54 mL, 2.30 mmol, 7.5 equiv). The reaction was stirred under argon and at 0°C for 2h, and then was allowed to warm to room temperature with stirring continued overnight (14h). The resulting yellow suspension was condensed to dryness with a rotary evaporator, and the residue was treated with water (20 mL). The mixture was extracted with EtOAc (3×30 mL). The combined organic layers were washed with brine (20 mL), and dried over anhydrous Na_2SO_4. Evaporation of solvent afforded the crude product, which was purified by CombiFlash silica gel chromatography (eluting with MeOH in DCM 0-7%) to give the product as yield solid (86.5 mg, yield 72.0%). NMR: ^1H (300 MHz, CD_3OD) δ (ppm) 1.27 (s, 3H), 1.33 (s, 3H), 4.50 (s, 1H), 6.61 (d, *J*=8.7 Hz, 2H), 7.24 (d, *J*=8.7 Hz, 2H), 7.58 (d, *J*=8.4 Hz, 2H), 7.85 (d, *J*=8.4 Hz, 2H); ^{13}C (75 MHz, CD_3OD) δ (ppm) 25.78, 26.07, 58.80, 70.74, 71.54, 76.68, 78.95, 84.60, 108.28, 114.21, 126.09, 127.55, 132.12, 133.82, 150.24, 167.83, 168.07; HRMS: calculated for $C_{22}H_{21}N_3O_4H^+$ 392.1610; found 392.1607 $[M+H]^+$.

Synthesis of LPC-040

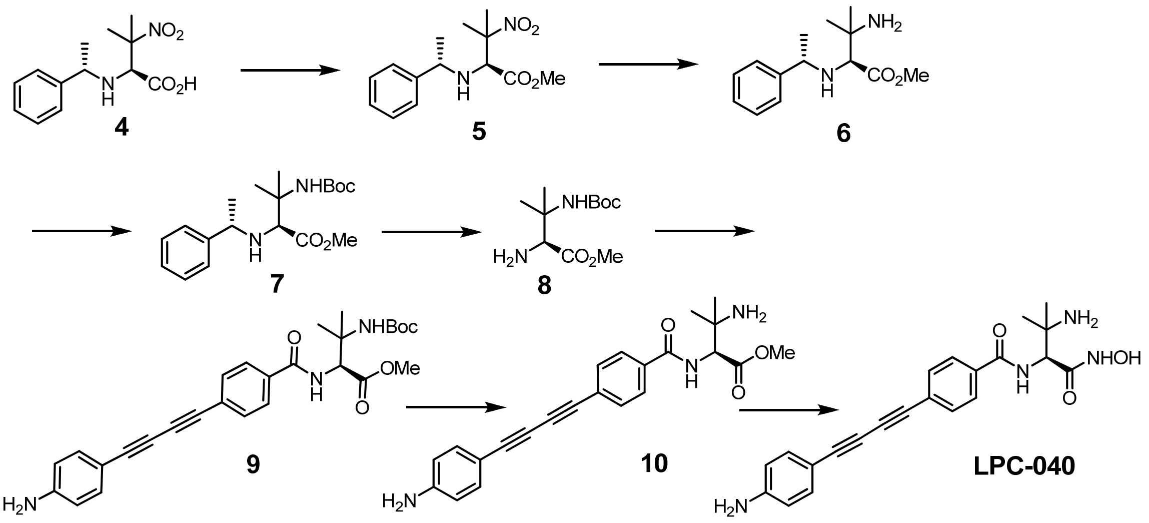

Compound **4**: (S)-3-Methyl-3-nitro-2-((S)-1-phenylethylamino)butanoic acid. 2-nitropropane (18.5 mL, 0.206 mmol, 1.02 equiv) and water (200 mL) were placed under argon in a 500 mL flask with stirring. Potassium hydroxide (13.60 g, 0.24 mmol, and 1.20 equiv) was added all at once. The flask was placed in a regulated oil bath and heated to 45°C. (S)-(-)-α-methybenzylamine (25.0 g, 0.206 mmol, 1.02 equiv) was added very quickly (very fast CO_2 absorption). The reaction mixture was maintained at 44-46°C with vigorous stirring, as glyoxylic acid (50% aq, 18.60 g, 0.202 mmol) was added slowly dropwise (60 min, slowest for the last one-third) via a syringe. The reaction mixture became cloudy, then clear, and when the solid began forming again the addition was slowed down. After completion of the addition, the reaction was stirred for an additional 3 h under argon at 35 °C, and stirred swiftly as 3M aq hydrochloric acid (152 mL, 0.46 mmol) was added dropwise (over 30 min). The resulting thick off-white suspension was stirred overnight (12 h) at rt. The cooled suspension was filtered with

suction, and the filter cake was rinsed with dilute aqueous HCl (0.2M, 0.5 L), water (0.5 L) and diethyl ether (0.125 L). The solid in the filter cake was dried by suction, and then dried under high vacuum for 3h at 50°C to get a slightly off-white power (27 g, 50% yield).

Purification of 3-methyl-3-nitro-(2(S)-(1(S)-phenylethyl-amino))-butyric acid **4**. 3M HCl (63 mL), water (0.72 L) and acetic acid (88 mL) were placed in an Erlenmyer flask and stirred well as it as immersed in a 60°C bath and warmed to 40-50°C (internal). When the solution was up to the temperature, 3-methyl-3-nitro-(2(s)-(1(s)-phenylethyl-amino))-butyric acid, dissolved in warm DMSO (dry, 40 mL) and acetic acid (25 mL), was added dropwise to the warm acid DMSO solution in the Erlenmeyer flask. The suspension was then filtered under vacuum and the filter cake was washed with ethyl ether (125 mL) and dried in vacuo. The solids then transferred to room temperature and dried under full vacuum for 12h (85% yield).

Second purification of 3-methyl-3-nitro-(2(S)-(1(S)-phenylethyl-amino))-butyric acid **4**. A solution of dilute aqueous hydrochloric acid (3.0M, 40 mL), water (500 mL) and acetic acid (80 mL) was placed in a 1L Erlenmeyer flask and stirred well as it was immersed in a 45-60°C bath and warmed to 40 °C (internal). A solution of 3-methyl-3-nitro-(2(s)-(1(s)-phenylethyl-amino))-butyric acid (5.10 g) in warm anhydrous DMSO (40 mL) and acetic acid (25 mL) was added dropwise to the Erlenmeyer flask. Upon complete addition, the suspension was stirred and placed in a 0°C bath to cool room temperature. The suspension was then filtered by suction and rinsed with dilute aqueous hydrochloric acid (0.2M, 500 mL), water (1 L), isopropanol (40 mL) and diethyl ether (125 mL). The filter cake was dried under vacuum for 14h. After that, the solid was dried at 50 °C under high vacuum for 3h to yield **4** as white powder (5.00 g, 98%). NMR ^{1}H (300 MHz, DMSO-d_6) δ (ppm) 1.21 (d, *J*=6.3 Hz, 3H), 1.43 (s, 3H), 1.45 (s, 3H), 3.62 (q, *J*=12.6 Hz, 1H), 7.17-7.31 (m, 5H); ^{13}C (75 MHz, DMSO-d_6) δ (ppm) 22.48, 24.05, 25.60, 57.14, 65.50, 98.69, 127.62, 127.81, 128.91, 144.85, 173.06; LC-MS: $[M+H]^+$267.

Compound **5**: Methyl (2S)-3-methyl-3-nitro-2-{[(1'S)-1'-phenylethyl]amino}butanoate. In an oven-dried 100 mL flask with a stir bar was charged with **4** (3.00 g, 11.3 mmol) and cesium carbonate (3.85 g, 11.82 mmol, 1.05 equiv) under argon with rapid stirring. Dimethylformamide (10 mL) was added rapidly and stirred for 10 min. After the reaction mixture was cooled to 0°C, iodomethane (0.81 g, 13.00 mmol, 1.15 equiv) was added dropwise over 15 min. The reaction mixture was stirred under argon and at 0°C for 1h, and was allowed to warm to ambient temperature with stirring continued for 12h. The reaction was washed with EtOAc and water into a separate funnel containing EtOAc (100 mL), water (20 mL) and 3.0 M aq hydrochloric acid. The organic layer was separated. And the aqueous layer was adjusted pH to 7-8 and extracted with EtOAc (2×100 mL). The combined organic phases were washed with 3% Li_2SO_4 (3×30 mL), half-saturated aq $NaHCO_3$ (30 mL) and brine (30 mL) and dried over anhydrous Na_2SO_4. The solvent was concentrated to give amber oil (2.93 g, 93% yield). NMR: ^{1}H (300 MHz, $CDCl_3$) δ (ppm) 1.33 (d, *J*=6.6 Hz, 3H), 1.49 (s, 3H), 1.54 (s, 3H), 2.06 (s, 1H), 3.60 (m, 1H), 3.75 (s, 3H), 7.21-7.35 (m, 5H); ^{13}C (75 MHz, $CDCl_3$) δ (ppm) 22.39, 24.02, 25.12, 52.55, 57.39, 65.20, 89.33, 127.43, 127.75, 128.64, 143.67, 172.67; LC-MS: $[M+H]^+$ 281.

Compound **6**: Methyl (2S)-3-amino-3-methyl-2-{[(1'S)-1'-phenylethyl]amino}butanoate**.** Compound **5** (2.83 g, 10.0 mmol) dissolved in anhydrous THF (30 mL) and glacial acetic acid (45 mL) along with activated powdered molecular sieves 4Å (3.4 g) was stirred gently for 3h under argon. The flask was then immersed in 0°C bath and stirred well for 20 min. To the cold

reaction mixture was added zinc dust (5.85 g, 90.0 mmol, 10.0 equiv). The mixture reaction was stirred at 0°C for 2h, and was allowed warm to ambient temperature with stirring continued for 16h. The mixture was then diluted with THF (40 mL), filtered through a celite pad and was washed with additional THF (100 mL). The filtrate was concentrated in vacuo to yield a slightly yellow oily solid. This material was dissolved in 3:1 chloroform/isopropanol (100 mL) and 0.25 EDTA solutions at pH 10.5-11 (60 mL). Additional 4M NaOH solution was added in portions to reach pH=10.5-11.0. The contents were thoroughly shaken, and the aqueous phase separated. The organic phase was then washed with 0.25 EDTA pH=10.5-11.0 (50 mL), brine (40 mL), dried (Na_2SO_4), filtered and evaporated under reduced pressure, followed with heptane (3×40 mL) and evaporation to yield a light-amber oil, which was purified by CombiFlash (eluting with MeOH in DCM 0-7%) to afford **6** as colorless oil (1.76g, 68% yield). NMR: ^{1}H (300 MHz, $CDCl_3$) δ (ppm) 1.00 (s, 1H), 1.05 (s, 1H), 1.32 (d, *J*=6.6 Hz, 3H), 2.74 (s, 1H), 3.56 (q, *J*=13.2 1H), 3.71 (s, 3H), 7.21-7.30 (m, 5H); ^{13}C (75 MHz, $CDCl_3$) δ (ppm) 25.68, 27.34, 27.90, 51.55, 51.81, 57.25, 68.54, 127.32, 128.58, 145.02, 176.05; LC-MS: $[M+H]^+$ 251.

Compound **7**: Methyl (2S)-3-{[(tert-butoxy)carbonyl]amino}-3-methyl-2-{[(1'S)-1'-phenylethyl]amino}butanoate. The compound **6** (1.70 g, 6.8 mmol) was dissolved in anhydrous THF (20 mL) under argon, and diisopropylethylamine (1.30 mL, 7.5 mmol, 1.10 equiv) was added to this solution. tert-Butylpyrocarbonate (1.78 g, 8.2 mmol, 1.20 equiv) was added. After stirring at rt for 16h, the reaction mixture was dissolved in EtOAc (100 mL), and washed with 0.3 M HCl (20 mL), half saturated $NaHCO_3$ solution, 14% aq. NH_4OH (30mL), brine (50 mL) and dried (Na_2SO_4), Solvent was removed under reduced pressure to yield the BOC-diamino-ester residue, which was purified by CombiFlash (eluting with EtOAc in DCM 0-5%) to afford it as viscous oil (2.10 g, 88% yield). NMR: ^{1}H (300 MHz, $CDCl_3$) δ (ppm) 1.27-1.37 (m, 18H), 1.99 (br s, 1H), 3.16 (br s, 1H), 3.55 (q, *J*=20.1 Hz, 1H), 3.73 (s, 3H), 4.93 (br s, 1H), 7.22-7.30 (m, 5H); ^{13}C (75 MHz, $CDCl_3$) δ (ppm) 23.78, 24.17, 25.41, 28.59, 51.89, 54.37, 57.36, 66.07, 127.259, 127.47, 128.70, 144.72, 175.15; LC-MS: $[M+H]^+$ 351.1.

Compound **8**: Methyl (2S)-2-amino-3-{[(tert-butoxy)carbonyl]amino}-3-methylbutanoate. The compound **7** (0.50 g, 1.4 mmol) was dissolved in anhydrous THF (10 mL) in 50 mL flask and placed under argon. Palladium hydroxide catalyst (0.18 g, 20 wt%) was rapidly weight and added to the flask. The flask was then evacuated and the contents were subjected to hydrogenation under atmospheric pressure of hydrogen. After 2h, LC-MS showed that the reaction was complete. The resulting mixture was filtered through a celite pad, and the filtrate was condensed to dryness. The residue was purified by CombiFlash (eluting with 0-5% MeOH in DCM) to afford **8** as white solid (330 mg, 95% yield). NMR: ^{1}H (300 MHz, $CDCl_3$) δ (ppm) 1.30 (s, 3H), 1.37 (s, 3H), 1.39 (s, 9H), 3.26 (br s, 2H), 3.71 (s, 3H), 3.87 (s, 1H), 4.95 (s, 1H); ^{13}C (75 MHz, $CDCl_3$) δ (ppm) 24.15, 24.23, 28.55, 52.28, 54.77, 60.55, 79.77, 155.26, 173.41; LC-MS: $[M+H]^+$ 247.3.

Compound **9**: Methyl (2S)-2-({4'-[4'-(4'-aminophenyl)buta-1',3'-diyn-1'-yl]phenyl} formamido)-3'-{[(tert-butoxy)carbonyl]amino}-3-methylbutanoate. To a stirred mixture of the compound 4-((4-aminophenyl)buta-1,3-diyn-1-yl)benzoic acid [3] (141mg, 0.54 mmol) and compound **8** (200 mg, 0.65 mmol, 1.20 equiv) in anhydrous DMF (5 mL) was added N-ethyl-N'-(3-dimethylaminopropyl) carbodiimide hydrochloride (124 mg 0.65 mmol, 1.20 equiv), 1-hydroxybenzotriazole (88 mg, 0.65 mmol, 1.20 equiv) at room temperature. The mixture was cooled with an ice-bath, and diisopropylethylamine (0.45 mL, 2.60 mmol, 5.00 equiv) was

added. The reaction mixture was stirred under argon and at 0°C for 1h, and was allowed to warm to temperature with the stirring continued for additional 14h. The resulting yellow solution was condensed to dryness with a rotary evaporator. And the residue was treated with water (20 mL), extracted with EtOAc (3×50 mL). The combined extracts were washed with brine (20 mL), and dried over anhydrous Na_2SO_4. Evaporation of the solvent was afforded the crude product, which was purified by flash chromatography (eluting with 0-2% MeOH in DCM) to afford the product (210 mg, 79% yield) as yellow solid. NMR: ^{1}H (300 MHz, CD_3OD) δ (ppm) 1.45 (m, 15H), 3.73 (s, 3H), 4.78 (d, *J*=6.9 Hz, 1H), 6.61 (d, *J*=8.1 Hz, 2H), 7.24 (d, *J*=8.4 Hz, 2H), 7.58 (d, *J*=8.1 Hz, 1H), 7.85 (d, *J*=8.1 Hz, 2H); ^{13}C (75 MHz, CD_3OD) δ (ppm) 24.00, 26.13, 27.66, 51.56, 53.90, 62.09, 70.92, 76.86, 79.09, 79.48, 84.71, 108.32, 114.25, 126.14, 127.40, 132.22, 133.62, 133.88, 150.17, 157.30, 167.46, 170.89; LC-MS: $[M+H]^+$490.

Compound **10**: Methyl (2S)-3-amino-2-({4'-[4'-(4'-aminophenyl)buta-1',3'-diyn-1'-yl] phenyl}formamido)-3-methylbutanoate. A flask containing compound **9** (200 mg, 0.41 mmol) was treated with TFA/DCM (1/6 mL) and stirred at 0°C under argon for 2h, then allowed to warm to ambient temperature with the stirring continued for additional 10h. The reaction mixture was concentrated by rotary evaporation to give a yellow solid. The residue was treated with water (10 mL), 1N NaOH was added dropwise to the mixture solution until pH=11, then extracted with EtOAc (3×50 mL). The combined extracts were washed with water (20 mL) and brine (20 mL), and dried over anhydrous Na_2SO_4. Evaporation of the solvent afforded the crude products, which was purified by flash chromatography (eluting with 0-5% MeOH in DCM) to afford the product (144 mg, 90% yield) as yellow solid. NMR: ^{1}H (300 MHz, CD_3OD) δ (ppm) 1.21 (s, 3H), 1.25 (s, 3H), 3.77 (s, 3H), 4.64 (s, 1H), 6.62 (d, *J*=8.7 Hz, 2H), 7.24 (d, *J*=8.7 Hz, 2H), 7.58 (d, *J*=8.4 Hz, 2H), 7.83 (d, *J*=8.4 Hz, 2H); ^{13}C (75 MHz, CD_3OD) δ (ppm) 26.22, 26.71, 51.49, 52.00, 61.67, 70.72, 76.66, 78.95, 84.60, 108.25, 114.20, 126.08, 127.66, 132.07, 133.82, 150.26, 168.39, 171.48; LC-MS: $[M+H]^+$390.

Compound **LPC-040**: (2S)-3-amino-2-({4'-[4'-(4'-aminophenyl)buta-1',3'-diyn-1-yl] phenyl}formamido)-N-hydroxy-3-methylbutanamide. To an ice-cold solution of the compound **10** (130 mg, 0.33 mmol) dissolved in anhydrous MeOH (1.5 mL) and THF (1.5 ml) was added hydroxylamine hydrochloride (116.0 mg, 1.67 mmol 5.0 equiv), followed by 25% sodium methoxide in methanol solution (0.59 mL, 2.51 mmol, 7.5 equiv). The reaction was stirred under argon and at 0°C for 2h, was allowed to warm to room temperature with stirring continued overnight (18 hours). The resulting yellow suspension was concentrated to dryness with a rotary evaporator. And the residue was treated with water (20mL). The mixture was extracted with 1-butanol (3×30 ml), washed with brine (20 ml), and dried over anhydrous Na_2SO_4. Evaporation of solvent afforded the crude products, which was purified by CombiFlash (eluting with MeOH in DCM 0-12%) to give it as white solid (89.0 mg, yield 68%). NMR: ^{1}H (300 MHz, CD_3OD) δ (ppm) 1.28 (s, 3H), 1.30 (s, 3H), 4.52 (s, 1H), 6.62 (d, *J*=8.4 Hz, 2H), 7.24 (d, *J*=8.4 Hz, 2H), 7.58 (d, *J*=8.4 Hz, 2H), 7.87 (d, *J*=8.1 Hz, 2H); ^{13}C (75 MHz, CD_3OD) δ (ppm) 25.21, 25.83, 53.36. 57.58, 70.72, 76.73, 78.93, 84.65, 108.26, 114.20, 126.17, 127.67, 132.07, 133.71, 133.81, 150.25, 167.53, 168.08; HRMS: calculated for $C_{23}H_{22}N_3O_3H^+$ 391.1770; found 391.1767 $[M+H]^+$.

Synthesis of LPC-058

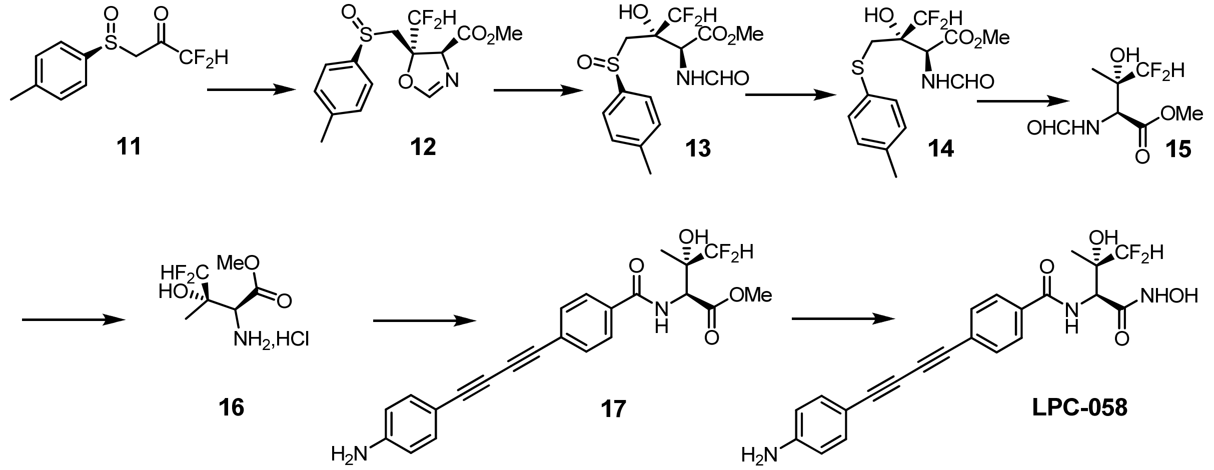

Compound **11**: (*S*)-1,1-difluoro-3-(*p*-tolylsulfinyl)propan-2-one. To a solution of diisopropylamine (16.21 mmol) in anhydrous THF (30 mL) was added BuLi (1.70 in hexane, 9.15 mL, 15.56 mmol, 1.20 equiv) at -78°C. After 30 min, a solution of S(-)-methyl *p*-toluyl sulfoxide in anhydrous THF (10 mL) was added dropwise. Then the reaction mixture was stirred at -78°C for 30 min. Ethyl fluoroacetate (15.56 mmol, 1.20 equiv) was added dropwise. The reaction mixture was stirred at -78°C for 2h under argon. Then the mixture was quenched with saturated NH_4Cl (100 mL), extracted with EtOAc (3×100 mL). The combined extracts were washed with water (100 mL), brine (100 mL) and dried (anhydrous Na_2SO_4). The crude product was purified by CombiFlash (eluting with EtOAc in hexane 0-50%) to give **11** as a white solid (88%). NMR: ^{1}H (300 MHz, $CDCl_3$) δ (ppm) 2.40 (s, 3H), 3.87 (dd, J=3.3, 13.2 Hz, 1H), 3.94 (dd, J=2.7, 13.8 Hz, 1H), 4.70 (s, 1H), 4.86 (s, 1H), 7.33 (d, J=7.8 Hz, 2H), 7.51 (d, J=8.4 Hz, 2H); ^{19}F (300 MHz, $CDCl_3$) δ (ppm) -128.00, -132.84 (ABq, d, 2F, J_{HF}=9 Hz, J_{FF}=58 Hz); ^{13}C (75 MHz, $CDCl_3$) δ (ppm) 1.69, 63.86, (84.72, 87.19), 124.21, 130.51, 139.24, 142.83, (197.25, 197.52); LC-MS $[M+H]^+$ 215.0.

Compound **12**: Methyl (4*S*,5*S*)-5-(difluoromethyl)-5-(((*S*)-*p*-tolylsulfinyl)methyl)-4,5-dihydro-oxazole-4-carboxylate. To a suspension of **11** (11.2 mmol) in anhydrous diethyl ether (50 mL) was added Cu_2O (0.16 g, 1.12 mmol, 0.10 equiv) at 0°C under argon. The reaction mixture was added with methyl isocyanoacetate (2.21 g, 2.00 equiv) dropwise. The reaction mixture was stirred at 0°C for 2h, and was allowed to warm to room temperature for 14h. The resulting mixture was concentrated to dryness. The crude product was purified by CombiFlash systerm(eluting with EtOAc in hexane 0-60%) to give the **12** as yellow oil (40-50% yield). NMR: ^{1}H (300 MHz, $CDCl_3$) δ (ppm) 2.37 (s, 3H), 2.84-2.90 (m, 1H), 3.03 (d, J=13.5 Hz, 1H), 3.68 (s, 3H), 4.60 (dd, J=10.8, 45.6 Hz, 1H), 4.91 (s, 1H), 5.11 (ddd, J=1.2, 10.8, 48.3 Hz, 1H), 7.02 (s, 1H), 7.29 (d, J=7.5 Hz, 2H), 7.46 (d, J=8.1 Hz, 2H); ^{19}F (300 MHz, $CDCl_3$) δ (ppm) -133.40, -135.22 (ABq, d, 2F, J_{HF}=59 Hz, J_{FF}=312 Hz); ^{13}C (75 MHz, $CDCl_3$) δ (ppm) 21.61, 52.96, 60.47, (71.41, 71.49), 82.24, 84.62, (85.33, 85.57), 124.11, 130.48, 140.38, 142.52, 155.80, 169.20; LC-MS:[314.1 M+H]$^+$.

Compound **13**: Methyl (2*S*,3*S*)-4,4-difluoro-2-formamido-3-hydroxy-3-(((*S*)-*p*-tolylsulfinyl) methyl)butanoate**.** To a solution of **12** (1.20 g, 3.78 mmol) in $CHCl_3$ (1 mL) was added deionized water (30 mL). The reaction mixture was stirred at room temperature under argon for 24h. Then the mixture was diluted with DCM (100 mL), washed with water (40 mL), brine (40 mL) and dried (anhydrous Na_2SO_4). The crude product was purified by CombiFlash (eluting with MeOH in DCM 0-5 %) to give **13** as white solid (yield 94%). NMR: ^1H (300 MHz, $CDCl_3$) δ (ppm) 2.37 (s, 3H), 3.12 (s, 2H), 3.73 (s, 3H), 4.50 (s, 1H), 4.66 (s, 1H), 5.01 (d, J=9.0 Hz, 1H), 5.40 (s, 1H), 7.28 (d, J=8.1 Hz, 2H), 7.40 (d, J=9.3 Hz, 1H), 7.51 (d, J=7.8 Hz, 2H), 8.20 (s, 1H); ^{19}F (300 MHz, $CDCl_3$) δ (ppm) -129.38, -133.12 (ABq, d, 2F, J_{HF}=58 Hz, J_{FF}=304 Hz); ^{13}C (75 MHz, $CDCl_3$) δ (ppm) 21.61, 53.25, 55.48, 62.04, (74.40, 74.64), (83.53, 85.87), 124.37, 130.41, 140.08, 142.27, 162.19, 169.92; LC-MS:$[M+H]^+$ 331.1.

Compound **14**: (2*S*,3*S*)-Methyl4,4-difluoro-2-formamido-3-hydroxy-3-((*p*tolylthio)methyl) butanoate. To a solution of **13** (3.4 mmol) in acetone (20 mL) was added sodium iodide (1.18 g, 7.84 mmol, 2.4 equiv) at -10°C under argon. A solution of trifluoroacetatic anhydride (0.75 mL in 2 mL acetone) was added dropwise over 8 min. The reaction mixture was stirred at -10°C for 9 min, and was concentrated to dryness. The residue was diluted with water (30 mL), extracted with EtOAc (3×50 mL). The combined organic layers were washed with water (30 mL), brine (30 mL) and dried (anhydrous Na_2SO_4). The crude product was purified by CombiFlash systerm (eluting with MeOH in DCM 0-3%) to give **14** as yellow oil (yield 88%). NMR: ^1H (300 MHz, $CDCl_3$) δ (ppm) 2.29 (s, 3H), 3.27-3.28 (m, 2H), 3.74 (s, 3H), 3.77 (d, J=4.5 Hz, 1H), 4.33 (s, 1H), 4.48 (s, 1H), 5.01 (d, J=9.3 Hz, 1H), 6.89 (d, J=8.4 Hz, 1H), 7.08 (d, J=7.8 Hz, 2H), 7.30 (d, J=7.2 Hz, 2H), 8.20 (s, 1H); ^{19}F (300 MHz, $CDCl_3$) δ (ppm) -131.14, -132.23 (ABq, d, 2F, J_{HF}=58 Hz, J_{FF}=300 Hz); ^{13}C (75 MHz, $CDCl_3$) δ (ppm) 21.24, 40.08, 53.16, 54.40, (75.01, 75.25), (82.64, 84.97), 130.18, 130.97, 132.14, 137.45, 161.73, 170.12; LC-MS: $[M+H]^+$ 316.1.

Compound **15**: (2*S*,3*S*)-Methyl 4,4-difluoro-2-formamido-3-hydroxy-3-methylbutanoate[4]. To a solution of **14** (2.82 mmol) in acetate buffer (pH 5.2) and MeOH (1:2, 90 mL) was added Raney Ni (suspension in methanol, 54 mL) followed by addition of sodium hypophosphite monohydrate (3.18 g in deionized water 12 mL) immediately. The reaction mixture was stirred at room temperature for 14h under argon. The mixture was filtered through a celite pad and was washed with MeOH (200 ml). The filtrate was concentrated to dryness. The residue was treated with water (50 mL), extracted with EtOAc (3×50 mL). The combined organic layers were washed with water (30 mL), 10% $NaHCO_3$ (2×30 mL), brine (30 mL), and dried (anhydrous Na_2SO_4). The crude product was purified by CombiFlash (eluting with MeOH in DCM 0-5%) to give **15** as white solid (yield 87%). NMR: ^1H (300 MHz, $CDCl_3$) δ (ppm) 1.24 (d, J=2.1 Hz, 3H), 3.73 (s, 3H), 4.17 (s, 1H), 4.32 (s, 1H), 4.41 (br, s, 1H), 4.74 (d, J=8.7 Hz, 1H), 7.23 (d, J=8.4 Hz, 1H), 8.17 (s, 1H); ^{19}F (300 MHz, $CDCl_3$) δ (ppm) -129.73, -134.13 (ABq, d, 2F, J_{HF}=59 Hz, J_{FF}=303 Hz); ^{13}C (75 MHz, $CDCl_3$) δ (ppm) 21.04, 52.87, 55.38, (72.79, 73.04), (85.37, 87.70), 162.35,170.63; LC-MS:$[M+H]^+$ 194.2.

Compound **16**: (2*S*,3*S*)-Methyl 2-amino-4,4-difluoro-3-hydroxy-3-methylbutanoate hydrochloride. Hydrochloric acid (0.188 mL, 2.26 mmol, 1.0 equiv) in methanol (1 mL) was added to a solution of **15** (2.26 mmol) in methanol (5 mL). The reaction mixture was stirred at room temperature for 14h. Then the mixture was concentrated to dryness. The residue was treated with water (30 mL), adjusted pH to 10 with 10% $NaHCO_3$, extracted with EtOAc (3×50 mL). The combined extracts were washed with water (30 mL), brine (30 mL) and dried

(anhydrous Na_2SO_4). Evaporation of the solvent was afforded **16** as white solid, which was carried to next step without further purification (yield 99%). NMR: ^{1}H (300 MHz, $CDCl_3$) δ (ppm) 1.06 (d, J=2.4 Hz, 3H), 3.57 (s, 1H), 3.72 (s, 3H), 4.26 (dd, J=9.3, 13.8 Hz, 1H), 4.52 (dd, J=4.44 (dd, J=9.3, 14.1 Hz, 1H); ^{19}F (300 MHz, D_2O) δ (ppm) -132.35, -134.49 (ABq, d, 2F, J_{HF}=58 Hz, J_{FF}=299 Hz); ^{13}C (75 MHz, $CDCl_3$) δ (ppm) 19.78, 19.84, 52.46, 57.49, (72.25, 72.48), (85.34, 87.66), 147.05; LC-MS: $[M+H]^+$ 166.0.

Compound **17**: (2*S*,3*S*)-Methyl 2-(4-((4-aminophenyl)buta-1,3-diyn-1-yl)benzamido)-4,4-difluoro-3-hydroxy-3-methylbutanoate. To a solution of 4-((4-aminophenyl)buta-1,3-diyn-1-yl)benzoic acid [3] (0.30 g, 1.25 mmol) in anhydrous DMF (5 mL) was added amine acid **16** (0.54 g, 1.31mmol, 1.05 equiv), EDC.HCl (0.26 g, 1.38 mmol, 1.2 equiv), HOBt (0.19 g, 1.38 mmol, 1.2 equiv) at room temperature under argon. The mixture was cooled to 0°C, DIEA (0.81 mL, 4.60 mmol, 4.00 equiv) was added. The reaction mixture was stirred at 0°C for 2h, then was allowed to warm to room temperature for 14h. The yellow solution was then concentrated to dryness. The residue was treated with water (30 mL), extracted with EtOAc (3×30 mL). The combined extracts were washed with water (50 mL), brine (50 mL), and dried (anhydrous Na_2SO_4). The crude product was purified by CombiFlash (eluting with MeOH in DCM 0-2.5 %) to give **17** as yellow solid (0.423 g, yield 80%). NMR: ^{1}H (300 MHz, CD_3OD) δ (ppm) 1.37 (d, J=2.1 Hz, 3 H), 3,76 (s, 3H), 4.267 (dd, J=9.3, 19.5 Hz, 1H), 4.42 (dd, J=9.3, 19.8 Hz, 1H), 4.81 (s, 1H), 6.61 (d, J=8.4 Hz, 2H), 7.24 (d, J=8.4 Hz, 2H), 7.56 (d, J=8.1 Hz, 2H), 7.82 (d, J=8.1 Hz, 2H); ^{19}F (400 MHz, CD_3OD) δ (ppm) -131.06, -137.61 (ABq, d, 2F, J_{HF}=60 Hz, J_{FF}=30 Hz); ^{13}C (75 MHz, CD_3OD) δ (ppm) 20.51, 51.69, 70.91, (71.94, 72.19), 76.86, 79.08, 84.73, (85.50, 87.83), 108.34, 114.27, 126.19, 127.20, 127.57, 132.18, 133.60, 133.89, 150.16, 168.18, 170.60; LC-MS:$[M+H]^+$ 437.1. HRMS $C_{23}H_{20}F_2N_2O_4$ caclated 426.1391 found: 426.1392.

Compound **LPC-058**: 4-((4-Aminophenyl)buta-1,3-diyn-1-yl)-*N*-((2*S*,3*S*)-4,4-difluoro-3-hydroxy-1-(hydroxyamino)-3-methyl-1-oxobutan-2-yl)benzamide. To an ice-cold solution of **17** (0.40 g, 0.94 mmol) dissolved in anhydrous MeOH (2 mL) and THF (2 mL) was added hydroxylamine hydrochloride (0.33 g, 4.69 mmol, 5.0 equiv) followed by 25% sodium methoxide in methanol solution (1.60 mL, 7.05 mmol, 7.5 equiv). The reaction mixture was stirred under argon and at 0°C for 2h, and allowed to warm to ambient temperature with the stirring continued overnight (14h). The resulting yellow suspension was condensed to dryness with a rotary evaporator, and the residue obtained was treated water (50 mL) and saturated NH_4Cl (50 mL), and extracted with EtOAc (3×30 mL). The combined extracts were washed with water (50 mL), brine (50 mL), and dried over anhydrous Na_2SO_4. Evaporation of the solvent afforded the crude product, which was purified by CombiFlash (eluting with 0-5% MeOH in DCM) to afford the title compound as yellow solid (0.33, 82% yield). NMR: ^{1}H (300 MHz, CD_3OD) δ (ppm) 1.36 (s, 3H), 4.73 (s, 1H), 5.80 (t, J=112.2 Hz, 1H), 6.61 (d, J=8.7 Hz, 2H), 7.24 (d, J=8.7 Hz, 2H), 7.57 (d, J=8.1 Hz, 2H), 7.82 (d, J=8.4 Hz, 2H); ^{19}F (400 MHz, CD_3OD) δ (ppm) -130.00, -137.91 (ABq, d, 2F, J_{HF}=60 Hz, J_{FF}=300 Hz); ^{13}C (75 MHz, CD_3OD) δ (ppm) 16.54, 54.87, 70.81, (72.60, 72.90, 73.19), 76.83, 78.97, 84.68, 108.36, (112.72, 115.96, 119.23), 114.26, 126.27, 127.53, 132.16, 133.47, 133.84, 150.16, 166.52, 167.51; LC/MS m/s $[M+H]^+$ 428.2. HRMS $C_{22}H_{19}F_2N_3O_4$ calculated 427.1344 found 427.1346.

Synthesis of $^{13}C/^{15}N$-labeled LPC-011 (compound 19)

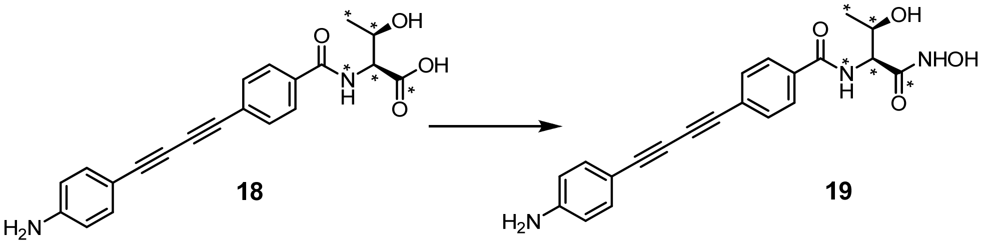

Compound **18**: (4-((4-aminophenyl)buta-1,3-diyn-1-yl)benzoyl)-L-threonine-[^{13}C4, ^{15}N]. To a stirred solution of the L-Threonine-[^{13}C4, ^{15}N] (37 mg, 0.3 mmol) in anhydrous DMF (2 mL) under an atmosphere of argon, was perfluorophenyl 4-((4-aminophenyl)buta-1,3-diyn-1-yl)benzoate [5] (128 mg, 0.3 mmol) followed by triethylamine (101 mg, 1 mmol). The mixture was stirred at room temperature for 16h. The reaction mixture was concentrated under reduced pressure and the residue obtained was purified on a CombiFlash silica gel chromatography system with MeOH/DCM (0 to 20% gradient) as eluent to afford the desired product as yellow solid (96 mg). LC-MS: $[M+1]^+$ 368.1; Calculated mass for $^{13}C_4C_{17}H_{18}{}^{15}NNO_4$: 367.1

Isotope labeled **LPC-011** (compound **19**): 4-((4-aminophenyl)buta-1,3-diyn-1-yl)-N-((2S,3R)-3-hydroxy-1-(hydroxyamino)-1-oxobutan-2-yl)benzamide-[13C4, 15N]. To a stirred solution of compound **18** (95 mg, 0.26 mmol) in anhydrous DMF (3 mL), at room temperature under argon, was added hydroxylamine hydrochloride (70 mg, 1 mmol) and NEt3 (202 mg, 2 mmol), followed by PyBOP (203 mg, 0.39 mmol). The reaction mixture was stirred at 16 h, and concentrated in vacuo to dryness. The residue was partitioned between water (10 mL) and EtOAc (20 mL) with vigorous stirring. The organic layer washed with water (2x10 mL), brine (10 mL), and dried (anhydrous Na_2SO_4). The crude product was purified by CombiFlash silica gel chromatography with EtOAc/DCM (0 to 100% gradient) as eluant to afford isotope labeled **LPC-011 (**compound **19)** as a yellow solid (30 mg). LC-MS: $[M+1]^+$ 383.1; Calculated mass for $^{13}C_4C_{17}H_{19}{}^{15}NN_2O_4$: 382.1. NMR: ^{1}H (400 MHz, CD_3OD) δ (ppm) 1.25 (br d, J=128 Hz, 3H), 4.20 (br d, J=144 Hz, 1H), 4.43 (br d, J=140 Hz, 1H), 6.63 (d, J=8.8 Hz, 2H), 7.25 (d, J=8.8 Hz, 2H), 7.59 (d, J=8.8 Hz, 2H), 7.89 (d, J=8.8 Hz, 2H).

Synthesis of $^{13}C/^{15}N$-labeled LPC-023 (compound 21)

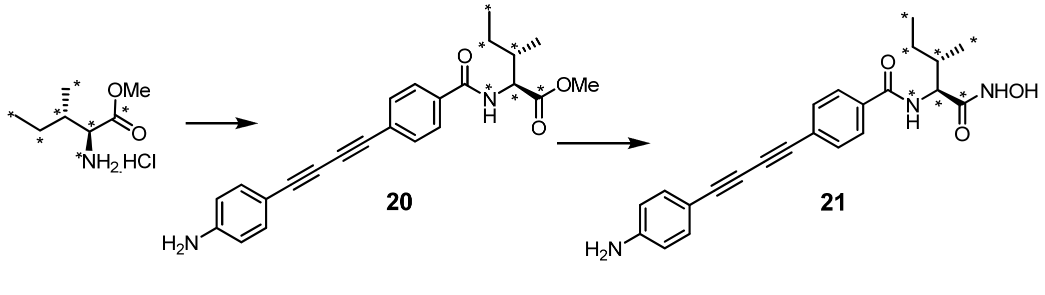

Compound **20**: Methyl (4-((4-aminophenyl)buta-1,3-diyn-1-yl)benzoyl)-L-isoleucinate-[^{13}C6, ^{15}N]. Methyl ester of L-Isoleucine-[^{13}C6, ^{15}N] was used to prepare compound **20** employing the procedure used for the corresponding unlabeled analog, compound **1**. The desired product was obtained as yellow solid after purification by CombiFlash silica gel chromatography

with EtOAc/DCM (0 to 100% gradient) as eluent. LC-MS: $[M+1]^+$ 396.2; Calculated mass for $^{13}C_6C_{18}H_{24}{}^{15}NN_2O_3$: 395.2

Isotope-labeled **LPC-023** (compound **21**): 4-((4-aminophenyl)buta-1,3-diyn-1-yl)-N-((2S,3S)-1-(hydroxyamino)-3-methyl-1-oxopentan-2-yl)benzamide-[^{13}C6, ^{15}N]. To a stirred solution of compound **20** (83 mg, 0.21 mmol) in MeOH (2 mL) and THF (1 mL), at 0-5°C under argon, was added 50% aqueous hydroxylamine (0.15 mL, 2.4 mmol), followed by addition of solid $LiOH.H_2O$ (18 mg, 0.42 mmol). The reaction mixture was allowed to stir for 16h with gradual warming to RT. The reaction was quenched with the addition of AcOH (0.1 mL) and the contents were partitioned between saturated NH_4Cl (5 mL) and EtOAc (10 mL). The organic layer was washed with water (10 mL) and brine (5 mL) and dried (anhydrous Na_2SO_4). The crude product was purified by CombiFlash silica gel chromatography with MeOH/DCM (0 to 10% gradient) as eluent to afford isotope-labeled **LPC-023** (compound **21)** as a yellow solid (58 mg). LC-MS: $[M+1]^+$ 397.2; Calculated mass for $^{13}C_6C_{17}H_{23}{}^{15}NN_2O_3$: 396.1. NMR: ^{1}H (400 MHz, CD_3OD) δ (ppm) 0.75-0.82 (m, 3H), 1.10-1.14 (m, 3H), 1.3-1.5 (m, 1H), 1.7-1.9 (m, 1H), 2.08-2.22 (m, 1H), 4.26 (br d, J=136 Hz, 1H), 6.62 (d, J=8.8 Hz, 2H), 7.24 (d, J=8.8 Hz, 2H), 7.57 (d, J=8.8 Hz, 2H), 7.82 (d, J=8.8 Hz, 2H).

Synthesis of LPC-083

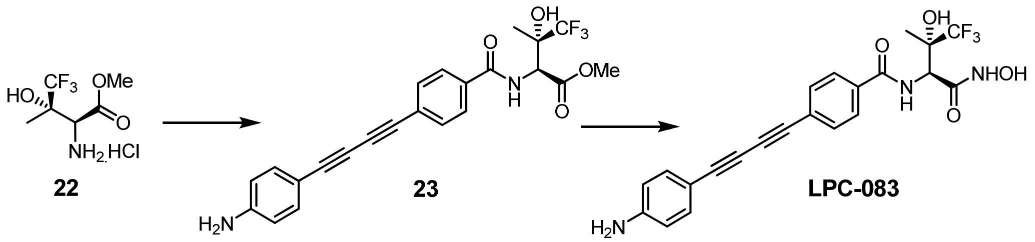

Compound **22**: Methyl (2S,3S)-2-amino-4,4,4-trifluoro-3-hydroxy-3-methylbutanoate hydrochloride. Compound **22** was prepared from (2S,3S)-2-amino-4,4,4-trifluoro-3-hydroxy-3-methylbutanoic acid [6] using the method as described for compound **2.** LC-MS: $[M+1]^+$ 202.1; Calculated mass for $C_6H_{10}F_3NO_3$: 201.1. NMR: ^{1}H (400 MHz, DMSO-d_6) δ (ppm) 1.40 (s, 3H), 3.76 (s, 3H), 4.13 (s, 1H), 7.47 (br s, 1H), 8.77 (br, 3H). ^{19}F (400 MHz, DMSO-d_6) δ (ppm) -79.2. ^{13}C (176 MHz, DMSO-*d*$_6$) δ (ppm) 16.5, 53.1, 55.4, 72.6 (q), 125.0 (q), 166.5.

Compound **23**: Methyl (2S,3S)-2-(4-((4-aminophenyl)buta-1,3-diyn-1-yl)benzamido)-4,4,4-trifluoro-3-hydroxy-3-methylbutanoate. Compound **23** was prepared using standard EDC-mediated amide coupling conditions. The desired product was obtained as yellow solid after purification by CombiFlash silica gel chromatography with EtOAc/DCM (0 to 100% gradient) as eluent. LC-MS: $[M+1]^+$ 445.1; Calculated mass for $C_{23}H_{19}F_3N_2O_4$: 444.1. NMR: ^{1}H (400 MHz, CD_3OD) δ (ppm) 1.52 (s, 3H), 3.79 (s, 3H), 5.01 (s, 1H), 6.62 (d, J = 8.8 Hz, 2H), 7.25 (d, J = 8.8 Hz, 2H), 7.59 (d, J = 8.8 Hz, 2H), 7.81 (d, J = 8.8 Hz, 2H). ^{19}F (400 MHz, CD_3OD) δ (ppm) -81.2. ^{13}C (176 MHz, Methanol-*d*4) δ (ppm) 19.7, 53.2, 57.9, 72.0, 75.0, 78.1, 80.2, 86.0, 109.6, 115.5, 127.6, 127.9, 128.9, 133.5, 134.9, 135.2, 151.6, 169.1, 170.7.

LPC-083: 4-((4-aminophenyl)buta-1,3-diyn-1-yl)-N-((2S,3S)-4,4,4-trifluoro-3-hydroxy-1-(hydroxylamino)-3-methyl-1-oxobutan-2-yl)benzamide. **LPC-083** was prepared from compound **23** employing the method described for the preparation of compound **21**. The desired product was obtained as yellow solid after purification on a by CombiFlash silica gel chromatography with MeOH/DCM (0 to 10% gradient) as eluent. LC-MS: $[M+1]^+$ 446.1; Calculated mass for $C_{22}H_{18}F_3N_3O_4$: 445.1. NMR: ^1H (400 MHz, DMSO-d_6) δ (ppm) 1.40 (s, 3H), 4.85 (d, J = 9.2 Hz, 1H), 5.87 (s, 2H), 6.47 (s, 1H), 6.55 (d, J = 8.4 Hz, 2H), 7.27 (d, J = 8.4 Hz, 2H), 7.68 (d, J = 8.4 Hz, 2H), 7.86 (d, J = 8.4 Hz, 2H), 8.35, (d, J = 9.2 Hz, 1H), 9.12 (s, 1H), 10.98 (s, 1H). ^{19}F (400 MHz, DMSO-d_6) δ (ppm) -78.6. ^{13}C (176 MHz, DMSO-*d*6) δ (ppm) 19.3, 53.5, 71.1, 73.4, 76.8, 79.2, 80.0, 85.8, 105.1, 113.6, 124.4, 127.8, 132.1, 133.9, 134.0, 150.9, 163.9, 164.8.

Supplementary References

1. Barb AW, Jiang L, Raetz CR, Zhou P. Structure of the deacetylase LpxC bound to the antibiotic CHIR-090: Time-dependent inhibition and specificity in ligand binding. *Proc Natl Acad Sci USA* **104**, 18433-18438 (2007).

2. Liang X*, et al.* Syntheses, structures and antibiotic activities of LpxC inhibitors based on the diacetylene scaffold. *Bioorg Med Chem* **19**, 852-860 (2011).

3. Zhou P, Toone EJ. Ethynylbenzene derivatives. US (2013).

4. Nishide K, Shigeta Y, Obata K, Inoue T, Node M. Reductive desulfurization using the Raney nickel sodium hypophosphite combination system without racemization of a secondary alcohol. *Tetrahedron Letters* **37**, 2271-2274 (1996).

5. Lee CJ*, et al.* Structural Basis of the Promiscuous Inhibitor Susceptibility of Escherichia coli LpxC. *ACS chemical biology* **9**, 237-246 (2014).

6. Soloshonok VA, Avilov DV, Kukhar VP. Asymmetric Aldol Reactions of Trifluoromethyl Ketones with a Chiral Ni(II) Complex of Glycine: Stereocontrolling Effect of the Trifluoromethyl Group. *Tetrahedron* **52**, 12433-12442 (1996).
